# Supplementary material for: Self-reported disability in relation to mortality in rural Malawi: a longitudinal study of over 16 000 adults
Source: BMJ Open. 2020 Aug 27;10(8):e034802. doi: 10.1136/bmjopen-2019-034802 (PMC7454196; doi:10.1136/bmjopen-2019-034802)
Supplement: Supplementary data [file bmjopen-2019-034802supp001.pdf]

Table S1. Baseline characteristics of census participants and study participants at Round 1

	**Census**			**Study[1]**		
	Women	Men	Total	Women	Men	Total
	n=9779	n=8194	n=17973	n=7430	n=3425	n=10855
**Age group**						
**18-34**	5142 (52.6)	4472 (54.6)	9614 (53.5)	3813 (51.3)	1544 (45.1)	5357 (49.4)
**35-44**	1874 (19.2)	1598 (19.5)	3472 (19.3)	1476 (19.9)	711 (20.8)	2187 (20.1)
**45-54**	1105 (11.3)	934 (11.4)	2039 (11.3)	814 (11.0)	443 (12.9)	1257 (11.6)
**55-64**	781 (8.0)	553 (6.7)	1334 (7.4)	606 (8.2)	306 (8.9)	912 (8.4)
**65-69**	280 (2.9)	192 (2.3)	472 (2.6)	213 (2.9)	105 (3.1)	318 (2.9)
**70-74**	197 (2.0)	142 (1.7)	339 (1.9)	173 (2.3)	101 (2.9)	274 (2.5)
**75-79**	200 (2.0)	154 (1.9)	354 (2.0)	164 (2.2)	96 (2.8)	260 (2.4)
**80+**	200 (2.0)	149 (1.8)	349 (1.9)	171 (2.3)	119 (3.5)	290 (2.7)
**Missing**	0	0	0	0	0	0
**Education level**						
**None**	379 (3.9)	104 (1.3)	483 (2.7)	296 (4.0)	69 (2.0)	365 (3.4)
**Some primary/completed primary**	6323 (65.1)	3961 (48.6)	10284 (57.6)	4975 (67.4)	1834 (53.7)	6809 (63.1)
**Some secondary/completed secondary**	2529 (26.1)	3442 (42.2)	5971 (33.4)	1757 (23.8)	1264 (37.0)	3021 (28.0)
**Tertiary**	477 (4.9)	649 (8.0)	1126 (6.3)	351 (4.8)	247 (7.2)	598 (5.5)
**Missing**	71	38	109	51	11	62
**Occupation**						
**not working**	1156 (12)	1497 (18.3)	2653 (14.9)	621 (8.4)	392 (11.5)	1013 (9.4)
**manual**	130 (1.3)	1086 (13.3)	1216 (6.8)	89 (1.2)	386 (11.3)	475 (4.4)
**farmer/fisherman**	7190 (74.3)	4639 (56.8)	11829 (66.3)	5716 (77.7)	2326 (68.1)	8042 (74.7)

**non-manual/ business/ professional**	1196 (12.4)	939 (11.5)	2135 (12)	928 (12.6)	310 (9.1)	1238 (11.5)
**Missing**	107	33	140	76	11	87
**Union status**						
**Not in a union**	3380 (34.6)	2635 (32.2)	6015 (33.5)	2339 (31.5)	869 (25.4)	3208 (29.6)
**In a union**	6391 (65.4)	5547 (67.8)	11938 (66.5)	5087 (68.5)	2553 (74.6)	7640 (70.4)
**Missing**	8	12	20	4	3	7
**BMI**						
**<18.5**	624 (7.2)	647 (9.6)	1271 (8.2)	482 (7.1)	298 (10.0)	780 (8.0)
**18.5-24.9**	5667 (65.0)	5434 (80.9)	11101 (71.9)	4344 (64.4)	2373 (79.8)	6717 (69.1)
**25-29.9**	1774 (20.3)	554 (8.2)	2328 (15.1)	1401 (20.8)	269 (9.0)	1670 (17.2)
**30+**	660 (7.6)	83 (1.2)	743 (4.8)	520 (7.7)	35 (1.2)	555 (5.7)
**Missing**	1054	1476	2530	683	450	1133
**Hypertension**						
**No hypertension**	6286 (85.8)	4639 (86.3)	10925 (86.0)	4871 (85.3)	2026 (82.4)	6897 (84.4)
**Hypertension**	1041 (14.2)	737 (13.7)	1778 (14.0)	837 (14.7)	434 (17.6)	1271 (15.6)
**Missing**	2452	2818	5270	1722	965	2687
**Diabetes**						
**No diabetes**	6400 (98.2)	4534 (98.2)	10934 (98.2)	4988 (98.3)	2057 (97.5)	7045 (98.1)
**Diabetes**	117 (1.8)	81 (1.8)	198 (1.8)	87 (1.7)	53 (2.5)	140 (1.9)
**Missing**	3262	3579	6841	2355	1315	3670
**HIV status**						
**Negative**	6804 (88.7)	5066 (91.0)	11870 (89.7)	5233 (88.4)	2206 (89.6)	7439 (88.7)
**Positive**	864 (11.3)	504 (9.0)	1368 (10.3)	686 (11.6)	257 (10.4)	943 (11.3)

**Missing**	2111	2624	4735	1511	962	2473
**Number of chronic diseases**						
**0**	4689 (77.4)	3219 (78.6)	7908 (77.9)	3633 (77)	1393 (73.5)	5026 (76.0)
**1**	1254 (20.7)	793 (19.4)	2047 (20.2)	1001 (21.2)	455 (24.0)	1456 (22.0)
**2**	107 (1.8)	84 (2.0)	191 (1.9)	82 (1.7)	45 (2.4)	127 (1.9)
**3**	5 (0.1)	2 (0.0)	7 (0.1)	4 (0.1)	2 (0.1)	6 (0.1)
**Missing**	3724	4096	7820	2710	1530	4240

1. Study participants defined as those with data on disability status

Table S2. Age-specific all-cause mortality rates of men with and without disability data[1]

	**With disability data**			**Without disability data**		
**Age group**	**Number of deaths**	**Person-years**	**Rate per 1000 PY**	**Number of deaths**	**Person-years**	**Rate per 1000 PY**
**18-49**	56	10198	5.49 (4.23-7.14)	29	5502	5.27 (3.66-7.86)
**50-69**	56	2207	25.37 (19.52-32.97)	18	3623	36.23 (22.82-57.50)
**70+**	61	972	62.74 (48.81-80.63)	12	65	184.23 (104.63-324.40)

1. p value for heterogeneity between men with and without disability data = 0.94

Table S3. Poisson regression analysis of effect of graded levels of self-reported disability on all-cause mortality

	**Number of deaths**	**Person-years**	**Model 1: adjusted for age only[1]**	**Model 2: adjusted for age, sex, & occupation[2]**	**Model 3: adjusted for age, sex, occupation, BMI, hypertension, diabetes, & HIV status[3,4]**

			RR (95% CI)	p- value[5]	RR (95% CI)	p- value[5]	RR (95% CI)	p- value[5]
**Any disability**								
**No difficulty**	48	13248	1		1		1	
**Some difficulty**	82	7537	1.31 (0.87-1.99)	0.12	1.51 (0.99-2.29)	0.10	1.50 (0.99-2.28)	0.09
**A lot of difficulty/ can't do at all**	50	1937	1.71 (1.02-2.86)		1.67 (0.98-2.85)		1.71 (1.01-2.90)	

1. Adjusted for age (continuous variable); excluding participants who were missing data on occupation, BMI, hypertension, diabetes, or HIV to allow comparison with Models 2 and 3
2. Adjusted for age (continuous variable), sex (male/female), and occupation (not working, manual work, farming/fishing, non-manual work); excluding participants who were missing data on BMI, hypertension, diabetes, or HIV to allow comparison with Models 1 and 3
3. Adjusted for age (continuous variable), sex (male/female), occupation (not working, manual work, farming/fishing, non-manual work), BMI (<18.5kg/m^2, 18.5-24.9kg/m^2, 25-29.9kg/m^2, 30+kg/m^2), hypertension (defined as a measured BP >140/90 or on antihypertensive medication), diabetes (defined as fasting blood sugar >7.0 or previous diagnosis of diabetes), and HIV status (defined as a positive HIV test or diagnosis ever)
4. Likelihood ratio test for heterogeneity in the association of disability and all-cause mortality by these factors: BMI p=0.08; hypertension p=0.67; diabetes p=0.41; HIV p=0.88
5. P value is a likelihood ratio test for difference

Table S4. Poisson regression analysis of effect of graded levels of self-reported disability on all-cause mortality by hypertension status (including unknown)[1,2]

	**No hypertension**			**Hypertension**			**Unknown hypertension status**		
	Deaths	Person-years	RR (95% CI)	Deaths	Person-years	RR (95% CI)	Deaths	Person-years	RR (95% CI)
**No disability**	48	14009	1	9	1286	1	27	7099	1
**Some difficulty**	59	6719	1.48 (0.99-2.20)	34	1790	1.72 (0.82-3.61)	30	2217	1.98 (1.14-3.41)
**A lot of difficulty / can't do at all**	30	1461	1.93 (1.16-3.20)	40	844	2.27 (1.07-4.81)	51	588	5.10 (2.94-8.83)

1. All models are controlled for age (continuous variable), sex, occupation (not working, manual work, farming/fishing, non-manual work)
2. p-value for heterogeneity = 0.03
