# Supplementary material for: Self-reported disability in relation to mortality in rural Malawi: a longitudinal study of over 16 000 adults
Source: BMJ Open. 2020 Aug 27;10(8):e034802. doi: 10.1136/bmjopen-2019-034802 (PMC7454196; doi:10.1136/bmjopen-2019-034802)
Supplement: Author's manuscript [file bmjopen-2019-034802.draft_revisions.pdf]

BMJ Open is committed to open peer review. As part of this commitment we make the peer review history of every article we publish publicly available.

When an article is published we post the peer reviewers' comments and the authors' responses online. We also post the versions of the paper that were used during peer review. These are the versions that the peer review comments apply to.

The versions of the paper that follow are the versions that were submitted during the peer review process. They are not the versions of record or the final published versions. They should not be cited or distributed as the published version of this manuscript.

BMJ Open is an open access journal and the full, final, typeset and author-corrected version of record of the manuscript is available on our site with no access controls, subscription charges or pay-per-view fees (http://bmjopen.bmj.com).

If you have any questions on BMJ Open's open peer review process please email info.bmjopen@bmj.com

BMJ Open

Self-reported disability in relation to mortality in rural Malawi: a longitudinal study of over 16000 adults

Journal:	*BMJ Open*
Manuscript ID	bmjopen-2019-034802
Article Type:	Original research
Date Submitted by the Author:	18-Oct-2019

Complete List of Authors:	Prynn, Josephine; University College London,<br>Dube, Albert; Malawi Epidemiology and Intervention Research Unit<br>Mkandawire, Joseph; Malawi Epidemiology and Intervention Research Unit<br>Koole, Olivier ; London School of Hygiene and Tropical Medicine Faculty of Infectious and Tropical Diseases<br>Geis, Steffen; Malawi Epidemiology and Intervention Research Unit; London School of Hygiene and Tropical Medicine Faculty of Infectious and Tropical Diseases<br>Mwaiyeghele, Elenaus; Malawi Epidemiology and Intervention Research Unit<br>Mwiba, Oddie; Malawi Epidemiology and Intervention Research Unit<br>Price, Alison; London School of Hygiene and Tropical Medicine Faculty of Infectious and Tropical Diseases<br>Kachiwanda, Lackson; Malawi Epidemiology and Intervention Research Unit<br>Nyirenda, Moffat; London School of Hygiene and Tropical Medicine Faculty of Infectious and Tropical Diseases; MRC/UVRI Uganda Research Unit On AIDS<br>Kuper, Hannah; London School of Hygiene and Tropical Medicine, Faculty of Epidemiology and Population Health<br>Crampin, Amelia; London School of Hygiene and Tropical Medicine Faculty of Infectious and Tropical Diseases; Malawi Epidemiology and Intervention Research Unit

Keywords:	EPIDEMIOLOGY, Hypertension < CARDIOLOGY, General diabetes < DIABETES & ENDOCRINOLOGY, HIV & AIDS < INFECTIOUS DISEASES, PUBLIC HEALTH

SCHOLARONE™
Manuscripts

I, the Submitting Author has the right to grant and does grant on behalf of all authors of the Work (as defined in the below author licence), an exclusive licence and/or a non-exclusive licence for contributions from authors who are: i) UK Crown employees; ii) where BMJ has agreed a CC-BY licence shall apply, and/or iii) in accordance with the terms applicable for US Federal Government officers or employees acting as part of their official duties; on a worldwide, perpetual, irrevocable, royalty-free basis to BMJ Publishing Group Ltd ("BMJ") its licensees and where the relevant Journal is co-owned by BMJ to the co-owners of the Journal, to publish the Work in this journal and any other BMJ products and to exploit all rights, as set out in our licence.

The Submitting Author accepts and understands that any supply made under these terms is made by BMJ to the Submitting Author unless you are acting as an employee on behalf of your employer or a postgraduate student of an affiliated institution which is paying any applicable article publishing charge ("APC") for Open Access articles. Where the Submitting Author wishes to make the Work available on an Open Access basis (and intends to pay the relevant APC), the terms of reuse of such Open Access shall be governed by a Creative Commons licence – details of these licences and which Creative Commons licence will apply to this Work are set out in our licence referred to above.

Other than as permitted in any relevant BMJ Author's Self Archiving Policies, I confirm this Work has not been accepted for publication elsewhere, is not being considered for publication elsewhere and does not duplicate material already published. I confirm all authors consent to publication of this Work and authorise the granting of this licence.

For peer review only

Self-reported disability in relation to mortality in rural Malawi: a longitudinal study of over 16000 adults

Corresponding Author

Dr Josephine E Prynn, University College London, London UK; Malawi Epidemiology and Intervention Research Unit (MEIRU), Lilongwe, Malawi
Josephineprynn@gmail.com
+447746427488
187 Jamaica St, London E1 0PE

Co-authors

Mr Albert Dube, Malawi Epidemiology and Intervention Research Unit (MEIRU), Lilongwe, Malawi

Dr Joseph Mkandawire, Malawi Epidemiology and Intervention Research Unit (MEIRU), Lilongwe, Malawi

Dr Olivier Koole, London School of Hygiene and Tropical Medicine, London, UK

Dr Steffen Geis, Malawi Epidemiology and Intervention Research Unit (MEIRU), Lilongwe, Malawi; London School of Hygiene and Tropical Medicine, London, UK; Institute for Medical Microbiology and Hygiene, Philipps University of Marburg, Marburg, Germany

Elenaus Myaiyeghele, Malawi Epidemiology and Intervention Research Unit (MEIRU), Lilongwe, Malawi

Mr Oddie Mwiba, Malawi Epidemiology and Intervention Research Unit (MEIRU), Lilongwe, Malawi

Dr Alison Price, London School of Hygiene and Tropical Medicine, London, UK

Mr Lackson Kachiwanda, Malawi Epidemiology and Intervention Research Unit (MEIRU), Lilongwe, Malawi

Prof Moffat Nyirenda, Malawi Epidemiology and Intervention Research Unit (MEIRU), Lilongwe, Malawi; London School of Hygiene and Tropical Medicine, London, UK; London School of Hygiene and Tropical Medicine, London, UK

Prof Hannah Kuper, London School of Hygiene and Tropical Medicine, London, UK

Prof Amelia C Crampin, Malawi Epidemiology and Intervention Research Unit (MEIRU), Lilongwe, Malawi**;** London School of Hygiene and Tropical Medicine, London, UK

Keywords or phrases
Disability; Epidemiology of chronic non communicable diseases; epidemiology; developing countries; mortality

Word count: 3045

For peer review only

Abstract

Objectives We investigated whether self-reported disability was associated with mortality in adults in rural Malawi.

Setting Karonga Health and Demographic Surveillance Site (HDSS), Northern Malawi

Participants All adults aged 18 and over residing in the HDSS were eligible to participate. During annual censuses in 2014 and 2015, participants were asked if they experienced difficulty in any of six functional domains and were classified as having disabilities if they reported “a lot of difficulty” or “can’t do at all” in any domain. Mortality data was collected until December 31st, 2017. 16748 participants (10153 women and 6595 men) were followed up for a median of 29 months.

Primary and secondary outcome measures We used Poisson regression to examine the relationship between disability and mortality adjusting for confounders. We assessed whether this relationship altered in the context of obesity, hypertension, diabetes, or HIV. We also evaluated whether people who died from NCDs were more likely to have reported disability than people dying from other causes as determined by verbal autopsy.

Results: At baseline 7.6% reported a disability and the overall mortality rate was 9.4/1000 person-years. People reporting disability had a mortality rate 2.70 times higher than those without. 44.4% of people who died from NCDs had disability compared to 16.1% of those who died from HIV.

Conclusions Self-reported disability predicts mortality at all adult ages in rural Malawi. Interventions to improve access to healthcare and other services are needed.

For peer review only

Strengths and Limitations of This Study

- We were able to use high-quality mortality data collected through a well-functioning health and demographic surveillance survey in a country without complete vital registration
- The large sample size allowed us to examine the relationship between disability and mortality over a relatively short follow-up period
- This is the first study to examine whether disability as defined by the Washington Group questions is predictive of mortality, so contributes substantially to the existing literature on this topic
- A limitation of the study is that we have considerable missing data in chronic health states (BMI, hypertension, diabetes, HIV) which limits our understanding of how these are related to disability and mortality

For peer review only

Abbreviations

Body mass index (BMI)

Health and Demographic Surveillance Site (HDSS)

Non-communicable diseases (NCDs)

Sub-Saharan Africa (SSA)

Sustainable Development Goals (SDGs)

Introduction

Disability is a complex concept encompassing long-term physical, mental, intellectual or sensory impairments which, in interaction with personal and environmental factors, may limit people's participation in society on an equal basis with others.[1] The World Health Organisation (WHO) estimates that one billion people globally have a disability,[1] and the number is expected to rise further as the global population continues growing, life expectancy rises, and average age increases. People with disabilities are being "left behind" in terms of inclusion in education, employment, and social life,[1] and are at high risk of poverty.[2] Consequently, disability is recognised as an important development issue, and is explicitly referenced within five of the Sustainable Development Goals (SDGs).[3]

Disability is expected to be linked to increased mortality, through various pathways. Both disability and mortality are related to ageing and poverty, and so associations may arise through confounding.[1, 2] An underlying disease may also cause both disability and mortality, for instance diabetes can lead to impairments (e.g. visual impairment from diabetic retinopathy) and premature death. People with disabilities may find it more difficult to seek health-care due to a range of barriers,[1] thereby increasing their mortality risk. They may also have unhealthier behaviours, on average, regarding smoking, physical activity, and diet, increasing their vulnerability to obesity, non-communicable diseases (NCDs) and mortality.[4] There is also evidence that people with disabilities are more vulnerable to HIV and other infectious diseases, due to their marginalised position in society.[5] Certain impairments, such as mobility or cognitive impairments, can increase frailty, depression and functional difficulties, all linked to increased mortality.[6-8] Data are, however, lacking on the link between disability and mortality, particularly from low- and middle-income settings, where over 80% of people with disabilities reside.[9] The debate has raged as to how disability should be defined and measured, yet scales are needed to estimate the prevalence and impacts of disability, and to allow international data comparisons. Consensus is growing on the use of the Washington Group (WG) Short Set to collect Disability Statistics,[10] which will improve data comparability. The WG questions focus on difficulties in functioning in 6 different domains related to activities (e.g. walking) and participation (e.g. performing usual activities).

The objective of this study was to investigate the relationship between self-reported disability and mortality among adults in rural Malawi. A secondary objective was to assess whether the effect of disability on mortality varies with co-existence of obesity or a chronic disease (hypertension, diabetes, or HIV), and by cause of death.

For peer review only

Methods

Setting and data collection

The Karonga Health and Demographic Surveillance Site (HDSS) in Northern Malawi comprises a population of around 40,000 individuals, which is largely representative of the rural Malawian population in terms of age and sex structure.[11, 12] Census information is collected on the population annually, along with continuous reporting of births, deaths, and migration by community key informants. Verbal autopsy is done after every death using a semi-structured interview of a family member using an adaptation of a WHO instrument.[13] Two clinically trained reviewers independently assign cause of death based on this. In case of disagreement, a third reviewer arbitrates.

Since 2014, the WG short set questions on disability have been added to the annual census questionnaire, alongside the existing questions on demographic, health, and social indicators. The disability questions were only asked if the participant was physically seen by the fieldworker, although they could be answered through a proxy, after obtaining written consent or assent. Therefore, no disability data was collected from anyone away from home on the day of the census.

The questions, translated into the local language of Chitumbuka, are:

- Do you have difficulty seeing, even if wearing glasses?
- Do you have difficulty hearing, even if using a hearing aid?
- Do you have difficulty walking or climbing steps?
- Do you have difficulty remembering or concentrating?
- Do you have difficulty (with self-care such as) washing all over or dressing?
- Using your usual (customary) language, do you have difficulty communicating, for example understanding or being understood?

In this analysis, we used data on adults aged 18 and over from the first two consecutive census rounds to include the disability questions. The first was in 2014-2015 (Round 1), the second in 2015-2016 (Round 2). Baseline disability status was taken from the Round 1 census data where possible. For participants with no disability data from Round

For peer review only

1, data from Round 2 was used. Anyone who moved into the HDSS between Rounds 1 and 2, or turned 18 between Rounds 1 and 2, and answered disability questions at Round 2 was also included. Other socio-demographic information was taken from the same interview as the disability data. Follow-up was undertaken until 31st December 2017.

Data on body mass index (BMI), hypertension, and diabetes were gathered from a survey of all adults performed within the HDSS population in 2013-2015 on prevalence of major NCDs and their risk factors.[14] In this screening, height and weight were measured twice, and the mean was used to calculate BMI. Participants were asked if they had previously been diagnosed with hypertension or diabetes, and whether they were taking any regular medication. Resting blood pressure was measured three times with 5 minutes in between, and a mean of the second and third measures were used. Blood was taken for plasma glucose measurement after at least 8 hours fasting. This data was collected a mean of 1.2 years prior to the disability data (maximum 3.5 years).

Data on HIV status was collated from numerous sources including an HIV serosurvey in 2011 and the 2013-2015 NCD survey. Data on new HIV diagnoses was also collected from consenting participants at government clinics within the HDSS. Participants were categorised as HIV-positive if they had ever reported a diagnosis of HIV or had a positive antibody test. They were categorised as negative for 4 years after a negative HIV-test, after which time their status was categorised as missing in case of a new infection in the interim.

Variables

Age was grouped into categories of 18-34, 35-44, 45-54, 55-64, 65-69, 70-74, 75-79, and 80+ years; narrower age bands were chosen at higher ages as self-reported disability is strongly associated with older age. Education level was defined as no education, primary (including partially-completed), secondary (including partially-completed), and tertiary. Occupation was categorised into not working (including unemployed, unable to work, and retired), manual work, farming or fishing, and non-manual work (including professional work). Participants were defined as in a union if they were married or co-habiting, and not if single, divorced, or widowed.

Categories of BMI were <18.5kg/m² (underweight), 18.5-24.9kg/m² (healthy weight), 25-29.9kg/m² (overweight), and 30+kg/m² (obese). Hypertension was defined as systolic blood pressure ≥140mmHg, diastolic blood pressure of ≥90mmHg, or use of antihypertensive medications. Diabetes was defined as fasting blood sugar ≥7.0mmol/L or a

For peer review only

self-reported diagnosis of diabetes. Disability was defined as answering “a lot of difficulty” or “can’t do at all” in any domain.

Cause of death among adults was broadly categorised into communicable disease, NCDs, maternal death, external (including injury and poisoning), and unspecifiable/other.

Statistical analysis

Prevalence of self-reported disability was calculated with 95% confidence intervals by socio-demographic and health characteristics. Poisson regression analysis was used to calculate mortality rate ratios, comparing people reporting “some difficulty” and “a lot of difficulty or can’t do at all” with “no difficulty” in each disability domain. For these analyses, individuals contributed exposure time during their residence in the HDSS from the date the disability survey was completed, until the earliest of 31st December 2017, death, or out-migration. Returning and repeat migrants only contributed person-years while resident in the HDSS.

Age and sex were included *a priori* in the adjusted model, and baseline occupation, education level, and union status were sequentially added to the model to check for confounding. Any variable that altered the rate ratio more than 10% was kept in the adjusted model. Each of obesity, hypertension, diabetes and HIV status at baseline were also added to the models to check for confounding or effect modification. We performed complete case analysis, so that any participants with missing data for any of the variables in the model were excluded. Sensitivity analyses, including an “unknown” category for BMI, hypertension, diabetes, and HIV were also performed.

Of people who died during the follow-up time, the proportion who reported disability prior to death was calculated and stratified by cause of death.

All significance tests were likelihood-ratio tests.

Ethical approval

Ethical approval for the HDSS census rounds and NCD survey was granted by the National Health Sciences Research Committee (NHSRC) (protocol numbers #419 and #1072 respectively), and by the London School of Hygiene and Tropical Medicine (LSHTM) (protocol numbers #5081 and #6303 respectively).

Patient and public involvement

For peer review only

MEIRU works closely with the community in which this research was conducted. Regular meetings with senior community-members take place to ensure that study objectives align with the priorities of the community, and that the methodology and procedures are appropriate and acceptable. Research findings are disseminated similarly.

Results

Of 17973 adult (≥18 years) residents seen at Round 1, 10855 (60.4%) responded to questions on disability. A further 5893 adults not included at Round 1 (because they were under 18 years, not living in the area at Round 1, or missed) provided disability data at Round 2, giving 16748 participants with baseline disability data (**Figure 1**).

Those without disability data were more likely to be younger (22.3% of 18-39-year-olds compared to 10.8% of those aged 40+) and male (29.4% of men compared to 9.5% of women). Participants with disability data had a median follow-up of 29 months (interquartile range 20-33 months). Migration out of the HDSS led to censoring of data in 2154 (21.9%) of participants. Individuals who migrated were more likely to be young and have higher educational attainment.

Table 1 shows that most participants were under age 35, and 76.1% of women and 62.5% of men were farmers or fishermen. The prevalence of obesity was higher in women than men (7.4% vs. 1.3%), whereas prevalence of hypertension, diabetes, and HIV were similar among men and women. Hypertension, diabetes, and HIV variables were not available for at least 30% of participants.

Prevalence of self-reported disability in one or more domain was 7.6% and strongly associated with increasing age (p test-for-trend <0.001). Adjusted for age, people with lower levels of education and those not working were more likely to report disability, and prevalence was higher among participants with obesity, hypertension, or diabetes. People living with HIV reported a similar prevalence of disability to those who were HIV negative.

There were 328 deaths in 36019 person-years of follow-up. The mortality rate was 9.1/1000 person-years (95% CI 8.2-10.1) and varied by sex (p <0.001); higher among men (12.9/1000 person-years; 95%CI 11.1-15.0) than women

For peer review only

(6.8/1000 person-years; 95% CI 5.8-8.0). Sensitivity analyses comparing all-cause mortality rates in men without disability data to those with disability data suggested no material difference stratified by age (**Table S1**).

Table 2 shows that compared with those reporting "no difficulty" in any disability domain, adults reporting "some difficulty", and "a lot of difficulty or can't do at all" had greater mortality (RR 1.39; 95% CI 1.03-1.88 and RR 2.70; 95% CI 1.91-3.82, respectively). Except for difficulty seeing and hearing, self-reported disability in any of the domains was strongly associated with increased mortality when adjusted for age, and the association remained when also adjusted for sex and occupation. For every domain, reporting disability ("a lot of difficulty" or "can't do at all") was associated with a higher mortality than answering "some difficulty". Disability in increasing numbers of domains was strongly associated with mortality. The relationship between any self-reported disability and all-cause mortality did not vary by sex or age group (**Table 3**); and none of BMI, hypertension, diabetes, or HIV were confounders or effect modifiers of the relationship (**Table S2**). Sensitivity analysis including a "missing" category for hypertension did show heterogeneity by hypertension status (p=0.03): the relationship between disability and mortality was stronger among those with unknown hypertension status (**Table S3**). Including a "missing" category for BMI, diabetes, and HIV did not show any heterogeneity.

Table 4 shows that 44.4% (95% CI 36.5-52.7%) of people who later died of a cause attributed to NCDs reported experiencing disability, compared to 16.1% (95% CI 8.9-27.5%) of those whose death was attributed to HIV, and 41.4% (95% CI 25.1-59.8%) of those whose death was attributed to a non-HIV communicable disease.

Discussion

Our findings show that self-reported disability in adults is strongly associated with increased all-cause mortality in rural Malawi. Furthermore, a stepwise increase in risk was observed with increasing levels of difficulties in functioning and numbers of domains affected. Individuals reporting difficulty in walking and self-care were particularly at risk. The co-existence of obesity or chronic disease did not materially alter estimates of effect, and the magnitude of the association was consistent across age- and sex- groups. People dying from NCDs were more likely to have been living with disability before death than those dying from HIV.

For peer review only

Our findings are similar to those observed elsewhere in sub-Saharan Africa (SSA). A study in Tanzanian adults aged 70+ also found a stepwise increase in mortality with increasing disability severity.[15] In South African adults aged 50+, severe functional disability was associated with a mortality rate ratio of 1.92 in women and 2.80 in men, compared to those with least functional disability.[16] In Malawi, Payne et al described an increasing mortality risk with increasingly severe disability among adults aged 45 and over, compared with those without disability.[17] None of these studies used the WG questions, however.

This association has also been observed in high-income countries (HIC),[18-21] even after adjusting for a wide range of comorbidities.[22-24] This suggests that the association between disability and mortality is not solely due to conditions predisposing to both disability and death. Nonetheless, contexts differ widely between high-resource settings and rural Malawi hence these findings may not be generalizable to our setting. In HIC, improved access to healthcare means that many more people live with treated chronic disease over many years, as opposed to in a low -income country (LIC) like Malawi, where people may be more likely to die early from disabling disease.[14, 25] Even access to safe water and sanitation services can be profoundly affected by disability in LIC including Malawi,[26, 27] which is an important risk factor for disease.

We observed a higher prevalence of disability in women than men at all ages, yet mortality rates were consistently higher in men than women across all adult age groups, irrespective of disability. This apparent “male-female health survival paradox” has been seen in a wide variety of settings, using a range of disability definitions.[15, 16, 23]

The population in Malawi is ageing, as in much of SSA.[28] As such, the very strong positive correlation between increasing age and prevalence of self-reported disability portends a growing prevalence of disability in the population in coming decades. The extent to which increasing life expectancy leads to an increased number of years lived with disability, or whether onset of disability will be delayed with a healthier life-course leading to increased life expectancy, is not yet understood.[29]

A major strength of our study is the available high-quality mortality data from a long-established HDSS, when vital registration is very limited in Malawi. [31] Moreover, the large sample size and low loss to follow-up allows meaningful mortality analyses despite a relatively short follow-up time. Disability is not a static state: people move considerably between disability states over time.[17, 30] Despite this, a one-off self-report of disability put

For peer review only

individuals at a much-increased risk of death in the following 2 years. As further disability and mortality data is collected with time, we will be able to examine mortality over a longer follow-up time and consider self-reported disability as a time-varying exposure in future analyses. Our study adds considerably to the very limited literature examining mortality with disability in this context and is the first to examine whether disability as defined by the WG questions is predictive of mortality.

Our study has several limitations. The WG questions do not capture a comprehensive picture of disability, for example common impairments such as affective disorders and chronic pain are not included. Furthermore, the use of self-report relies on participants having comparable interpretations of what constitutes "some difficulty" versus "a lot of difficulty". We have limited information on underlying health conditions in the population and our data on hypertension, diabetes, BMI, and HIV were not measured concurrently with disability. If participants were to change category in the intervening time misclassification could occur, but as these are all, in the main, long-term health states, it is unlikely that this will have affected a substantial number of participants. We had considerable missing data on hypertension, diabetes, and HIV status, and our data may have been more likely to capture positive cases than negative. For hypertension and diabetes, the data was missing mainly on younger, working men, who are likely to have lower prevalence than the general population.[31] For HIV status some data is from new diagnoses made at health centres, where people may present if they feel they are at risk of being HIV positive. This limits our understanding of the relationship of these conditions with disability and mortality. Our pragmatic approach to only collect disability reports on individuals found at home during our annual census means that the lower reports of disability and increased mortality in men compared to women seen in all age groups could be suspected to be due to a "healthy worker" effect as more men than women work far from the household in paid employment. However, we found that particularly among younger men, the mortality rate was very similar among those included in this analysis and those excluded.

Conclusions

Disability is commonly reported and likely to increase in Malawi in coming decades as the population ages and NCDs become increasingly prevalent. Self-reported disability is associated with significantly increased mortality, likely due partly to under-treatment of chronic conditions, and partly to barriers to healthy living faced by people living with

For peer review only

disability. While more research is urgently needed to help understand the mechanism behind this relationship, this needs to be concurrent with active interventions to improve access to healthcare and other services, particularly for people with disabilities, if the commitment to “leave no-one behind”[32] in the SDGs is to be realised.

References

1. World Health Organisation, *World Report on Disability*. 2011.
2. Banks, L.M., H. Kuper, and S. Polack, *Poverty and disability in low- and middle-income countries: A systematic review.* PLOS ONE, 2017. **12**(12): p. e0189996.
3. United Nations. *Sustainable Development Goals (SDGs) and Disability*. 16th April 2019]; Available from: https://www.un.org/development/desa/disabilities/about-us/sustainable-development-goals-sdgs-and-disability.html.
4. Hashemi, G., M. Wickenden, and H. Kuper, *SDGs, Inclusive Health and the path to Universal Health Coverage.* Disability and the Global South, 2017. **4**(1): p. 23.
5. De Beaudrap, P., M. Mac-Seing, and E. Pasquier, *Disability and HIV: a systematic review and a meta-analysis of the risk of HIV infection among adults with disabilities in Sub-Saharan Africa.* AIDS Care, 2014: p. 1-10.
6. Liljas, A.E.M., et al., *Self-reported vision impairment and incident prefrailty and frailty in English community-dwelling older adults: findings from a 4-year follow-up study.* J Epidemiol Community Health, 2017. **71**(11): p. 1053-1058.
7. Ribeiro, M.V., et al., *Association between visual impairment and depression in the elderly: a systematic review.* Arq Bras Oftalmol, 2015. **78**(3): p. 197-201.
8. Cimarolli, V.R. and D.S. Jopp, *Sensory impairments and their associations with functional disability in a sample of the oldest-old.* Qual Life Res, 2014. **23**(7): p. 1977-84.
9. World Health Organisation, *Concept Note: World Report on Disability and Rehabilitation*. 2007.
10. *Washington Group on Disability Statistics* Available from: http://www.washingtongroup-disability.com/.
11. Malawi National Statistical Office, *Population and Housing Census Results* 2008.
12. Dube, A., et al., *Profile: The Karonga Health and Demographic Surveillance System.* International Journal of Epidemiology, 2012. **41**(3): p. 676-685.
13. World Health Organisation. *Verbal autopsy standards: ascertaining and attributing causes of death*. 2018 14th July 2019]; Available from: https://www.who.int/healthinfo/statistics/verbalautopsystandards/en/.
14. Price, A.J., et al., *Prevalence of obesity, hypertension, and diabetes, and cascade of care in sub-Saharan Africa: a cross-sectional, population-based study in rural and urban Malawi.* The Lancet Diabetes & Endocrinology, 2018. **6**(3): p. 208-222.
15. Gray, W.K., et al., *Rates and predictors of three-year mortality in older people in rural Tanzania.* Archives of Gerontology and Geriatrics, 2016. **62**(Supplement C): p. 36-42.
16. Gómez-Olivé, F.X., et al., *Social conditions and disability related to the mortality of older people in rural South Africa.* International Journal of Epidemiology, 2014. **43**(5): p. 1531-1541.
17. Payne, C., J. Mkandawire, and H.-P. Kohler, *Disability Transitions and Health Expectancies among Adults 45 Years and Older in Malawi: A Cohort-Based Model.* PLoS Med, 2013. **10**(5): p. e100435.
18. Park, J.M., et al., *Disparities in mortality by disability: an 11-year follow-up study of 1 million individuals.* International Journal of Public Health, 2017. **62**(9): p. 989-996.
19. Björkenstam, C., et al., *Diagnosis-specific disability pension and risk of all-cause and cause-specific mortality – a cohort study of 4.9 million inhabitants in Sweden.* BMC Public Health, 2014. **14**(1): p. 1247.
20. Karlsson, N.E., et al., *Mortality in relation to disability pension: Findings from a 12-year prospective population-based cohort study in Sweden.* Scandinavian Journal of Public Health, 2007. **35**: p. 341-347.
21. Forman-Hoffman, V.L., et al., *Disability status, mortality, and leading causes of death in the United States community population.* Med Care, 2015. **53**(4): p. 346-54.

For peer review only

22. Hennessy, S., et al., *Disability Stage is an Independent Risk Factor for Mortality in Medicare Beneficiaries Aged 65 Years and Older.* PM&R, 2015. **7**(12): p. 1215-1225.
23. Pongiglione, B., et al., *Disability and all-cause mortality in the older population: evidence from the English Longitudinal Study of Ageing.* European Journal of Epidemiology, 2016. **31**(8): p. 735-746.
24. Majer, I.M., et al., *Mortality risk associated with disability: a population-based record linkage study.* Am J Public Health, 2011. **101**(12): p. e9-15.
25. Dewhurst, F., et al., *The prevalence of disability in older people in Hai, Tanzania.* Age Ageing, 2012. **41**(4): p. 517-23.
26. Groce, N., et al., *Water and sanitation issues for persons with disabilities in low- and middle-income countries: a literature review and discussion of implications for global health and international development.* Journal of Water and Health, 2011. **9**(4): p. 617.
27. White, S., et al., *A qualitative study of barriers to accessing water, sanitation and hygiene for disabled people in Malawi.* PLOS ONE, 2016. **11**(5).
28. Division, U.N.D.P., *World Population Prospects: The 2017 Revision*. 2017.
29. Beard, J.R., et al., *The World report on ageing and health: a policy framework for healthy ageing.* Lancet, 2016. **387**: p. 2145-54.
30. Prynn, J.E., et al., *Self-reported disability in rural Malawi: prevalence, incidence, and relationship to chronic disease [version 1; peer review: awaiting peer review].* Wellcome Open Research, 2019. **4**(90).
31. Crampin, A.C., et al., *Hypertension and diabetes in Africa: design and implementation of a large population-based study of burden and risk factors in rural and urban Malawi.* Emerg Themes Epidemiol, 2016. **13**: p. 3.
32. United Nations. *The Sustainable Development Goals Report*. 2016 16th August 2018]; Available from: https://unstats.un.org/sdgs/report/2016/leaving-no-one-behind.

Footnotes

Author Contributions

Conceptualisation: OK, AP, HK, ACC

Funding acquisition: MN, ACC

Design: AD, SG, AP

Project administration: AD, JM, EM, OM

Supervision of data collection: AD. OK, SG, EM, AP, LK

Data curation: OM

Analysis: JEP, ACC

Original draft preparation: JEP, HK, ACC

Review and editing of manuscript: JEP, AD, JM, OK, SG, EM, OM, AP, LK, MN, HK, ACC

Funding statement

For peer review only

This work was supported by the Wellcome Trust, grant number #098610

Competing interests

There were no competing interests for any author

Data Sharing Agreement

Data from the NCD survey is available from LSHTM Data Compass: Malawi Epidemiology and Intervention Research Unit Non-Communicable Disease Survey data, 2013–2017. https://doi.org/10.17037/DATA.0000096136. Data are available under the terms of the Creative Commons Attribution 3.0 International license (CC-BY 3.0).

Summary demographic datasets are publicly available through the INDEPTH iShare platform.

Longitudinal data (demographic surveillance episodes and linked rounds of disability questionnaires) cannot be sufficiently de-identified for public availability. Application may be made for access through the MEIRU director (mia.crampin@lshtm.ac.uk) or data scientist Chifundo Kanjala (chifundo.kanjala@lshtm.ac.uk). Those wishing to access the data will need to provide a brief proposal for what the data will be used for as a condition of access.

For peer review only

Table 1. Baseline characteristics of participants with baseline disability data and proportion with self-reported disability[1]

	Women			Men			Total		
	n	Number reporting disability (%)	p-value[2]	n	Number reporting disability (%)	p-value[2]	n	Number reporting disability (%)	p-value[2]
**Overall**	10153	857 (8.4)	-	6595	420 (6.4)	-	16748	1277 (7.6)	-
**Age group**									
18-34	5503	167 (3.0)	<0.001*	3378	73 (2.2)	<0.001*	8881	240 (2.7)	<0.001*
35-44	1914	103 (5.4)		1339	48 (3.6)		3253	151 (4.6)	
45-54	1100	122 (11.1)		786	55 (7.0)		1886	177 (9.4)	
55-64	773	116 (15.0)		486	64 (13.2)		1259	180 (14.3)	
65-69	258	54 (20.9)		175	25 (14.3)		433	79 (18.2)	
70-74	212	75 (35.4)		133	38 (28.6)		345	113 (32.8)	
75-79	189	94 (49.7)		138	45 (32.6)		327	139 (42.5)	
80+	204	126 (61.8)		160	72 (45.0)		364	198 (54.4)	
**Education level[3]**									
None	378	117 (31.0)	0.002*	98	27 (27.6)	0.06*	476	144 (30.3)	<0.001*
Some primary/completed primary	6603	632 (9.6)		3284	269 (8.2)		9887	901 (9.1)	
Some secondary/completed secondary	2633	82 (3.1)		2707	99 (3.7)		5340	181 (3.4)	
Tertiary	469	15 (3.2)		467	24 (5.1)		936	39 (4.2)	
**Occupation[4]**									
Not working	1013	202 (19.9)	<0.001	985	106 (10.8)	<0.001	1998	308 (15.4)	<0.001
Manual	143	5 (3.5)		745	28 (3.8)		888	33 (3.7)	
Farmer/fisherman	7637	567 (7.4)		4092	256 (6.3)		11729	823 (7.0)	
Non-manual/ business/ professional	1249	62 (5.0)		724	26 (3.6)		1973	88 (4.5)	
**Union status[5]**									
Not in a union	3381	479 (14.2)	<0.001	1933	97 (5.0)	0.001	5314	576 (10.8)	<0.001
In a union	6768	378 (5.6)		4659	323 (6.9)		11427	701 (6.1)	
**BMI (kg/m²)[6]**									
<18.5	669	61 (9.1)	0.005	601	57 (9.5)	0.38	1270	118 (9.3)	<0.001

For peer review only

18.5-24.9	6074	423 (7.0)		4847	263 (5.4)		10921	686 (16.3)	
25-29.9	1869	176 (9.4)		497	50 (10.1)		2366	226 (9.6)	
30+	689	90 (13.1)		80	10 (12.5)		769	100 (13.0)	
**Hypertension**[7]									
No hypertension	6069	416 (6.9)	0.69	3599	200 (5.6)	0.05	9668	616 (6.4)	0.05
Hypertension	1011	242 (23.9)		633	110 (17.4)		1644	352 (21.4)	
**Diabetes**[8]									
No diabetes	6189	535 (8.6)	0.22	3582	259 (7.2)	0.007	9771	794 (8.1)	0.008
Diabetes	109	25 (22.9)		69	19 (27.5)		178	44 (24.7)	
**HIV status**[9]									
Negative	6128	490 (8.0)	0.69	3070	218 (7.1)	0.60	9198	708 (7.7)	0.90
Positive	907	70 (7.7)		443	38 (8.6)		1350	108 (8.0)	
**Number of chronic diseases**[10,11]									
0	4243	261 (6.2)	0.77*	2054	102 (5.0)	0.002*	6297	363 (5.8)	0.02*
1	1166	170 (14.6)		620	91 (14.7)		1786	261 (14.6)	
2	100	19 (19.0)		69	14 (20.3)		169	33 (19.5)	
3	5	1 (20.0)		2	1 (50.0)		7	2 (28.6)	

1. Defined as answering “a lot of difficulty” or “can’t do at all” to at least one disability domain
2. p-value for difference using likelihood ratio test, values marked as * (age, education level, number of chronic diseases) p-value for trend. For all variables other than age group this is controlled for age (as a continuous variable)
3. Education level: No data on 70 women and 39 men
4. Occupation: No data on 111 women and 49 men
5. Union status: No data on 4 women and 3 men
6. BMI: No data on 872 women and 570 men
7. Hypertension: No data on 3073 women and 2363 men
8. Diabetes: No data on 3855 women and 2944 men
9. HIV status: No data on 3118 women and 3082 men
10. Chronic diseases included: hypertension, diabetes, HIV
11. Chronic diseases: No data on 4639 women and 3850 men (only participants with data on all 3 chronic diseases included)

For peer review only

Table 2. Poisson regression analysis of effect of graded levels of self-reported disability on all-cause adult mortality

	Number of deaths	Person-years	Model 1: adjusted for age only[1]		Model 2: adjusted for age, sex, & occupation[2]	
			RR (95% CI)	p- value[3]	RR (95% CI)	p-value[3]
**Difficulty seeing**						
No difficulty	165	28106	1	0.38		0.85
Some difficulty	107	6316	1.02 (0.79-1.34)		1.08 (0.82-1.41)	
A lot of difficulty/ can't do at all	49	1255	1.20 (0.84-1.73)		1.01 (0.7-1.46)	
**Difficulty hearing**						
No difficulty	296	33922	1	0.05		0.19
Some difficulty	39	1480	1.20 (0.85-1.71)		1.11 (0.78-1.58)	
A lot of difficulty/ can't do at all	13	275	1.79 (1.01-3.15)		1.51 (0.85-2.68)	
**Difficulty walking**						
No difficulty	157	30222	1	<0.001		<0.001
Some difficulty	79	4175	1.39 (1.04-1.88)		1.57 (1.15-2.13)	
A lot of difficulty/ can't do at all	85	1278	3.40 (2.48-4.66)		3.20 (2.27-4.51)	
**Difficulty remembering**						
No difficulty	204	29608	1	<0.001	1	<0.001
Some difficulty	79	5384	1.06 (0.81-1.39)		1.17 (0.89-1.54)	
A lot of difficulty/ can't do at all	37	650	2.67 (1.84-3.85)		2.55 (1.74-3.73)	
**Difficulty communicating**						
No difficulty	287	35235	1	<0.001	1	<0.001
Some difficulty	23	332	3.11 (2.01-4.8)		2.26 (1.44-3.53)	
A lot of difficulty/ can't do at all	10	82	8.81 (4.67-16.59)		5.33 (2.78-10.21)	
**Difficulty with self-care**						
No difficulty	234	33850	1	<0.001	1	<0.001

For peer review only

Some difficulty	47	1306	1.84 (1.33-2.56)		1.74 (1.24-2.45)	
A lot of difficulty/ can't do at all	38	503	3.75 (2.62-5.38)		3.14 (2.15-4.6)	
**Any disability**						
No difficulty	84	22197	1		1	
Some difficulty	122	10645	1.39 (1.03-1.88)	<0.001	1.61 (1.18-2.19)	<0.001
A lot of difficulty/ can't do at all	115	2839	2.70 (1.91-3.82)		2.65 (1.84-3.8)	
**Disability in multiple domains**						
No disability	206	32848	1		1	
Disability in 1 domain	51	2018	1.60 (1.15-2.21)		1.51 (1.08-2.11)	
Disability in 2 domains	31	543	2.39 (1.59-3.59)	<0.001	2.07 (1.35-3.17)	<0.001
Disability in 3 domains	19	120	3.71 (2.26-6.09)		3.06 (1.83-5.14)	
Disability in 4+ domains	14	79	5.64 (3.19-9.96)		4.33 (2.39-7.88)	

1. Controlled for age (continuous variable)
2. Controlled for age (continuous variable), sex, and occupation (not working, manual work, farming/fishing, non-manual work)
3. Likelihood ratio test for difference

For peer review only

Table 3. Poisson regression of disability[1] on all-cause adult mortality stratified by age group and sex[1]

Age group		Women: No. of deaths	Women: PY[3]	Women: Mortality rate per 1000 PY (95% CI)	Women: RR[4] (95%CI)	Women: p-value	Men: No. of deaths	Men: PY[4]	Men: Mortality rate per 1000 PY (95% CI)	Men: RR[3] (95% CI)	Men: p-value
**18-49**	No disability	40	16741	2.39 (1.75-3.26)	1	0.04	51	9870	5.17 (3.93-6.80)	1	0.10
	Disability	6	739	8.12 (3.65-18.09)	2.76 (1.16-6.58)		5	324	15.43 (6.42-37.06)	2.37 (0.94-5.97)	
**50-69**	No disability	25	3169	7.89 (5.33-11.67)	1	0.002	44	1952	22.54 (16.78-30.29)	1	0.06
	Disability	14	566	24.74 (14.65-41.77)	3.00 (1.55-5.79)		12	255	46.98 (26.68-82.73)	1.93 (1.02-3.65)	
**70+**	No disability	18	749	24.04 (15.15-38.15)	1	<0.001	29	636	45.62 (31.70-65.64)	1	0.009
	Disability	52	673	77.30 (58.90-101.45)	2.76 (1.61-4.75)		32	337	95.07 (67.23-134.44)	1.98 (1.19-3.30)	
**All**	No disability	83	20659	4.02 (3.24-4.98)	1	<0.001	124	12458	9.95 (8.35-11.87)	1	0.001
	Disability	72	1977	36.42 (28.91-45.88)	2.89 (1.20-4.19)		49	916	53.49 (40.42-70.77)	1.87 (1.30-2.71)	

1. Disability is defined as reporting “a lot of difficulty” or “can’t do at all” in any of the functional domains
2. p-value for heterogeneity between age-sex groups = 0.94
3. Person-years
4. Rate ratio, adjusted for age (as a continuous variable)

For peer review only

Table 4. Proportion of people living with disability prior to death, by physician assigned cause of death[1] using verbal autopsy

	No self-reported disability		Self-reported disability	
**Cause of death**	n	% (95% CI)	n	% (95% CI)
Communicable disease (except HIV)	17	58.6 (40.2-74.9)	12	41.4 (25.1-59.8)
HIV	52	83.9 (72.5-91.1)	10	16.1 (8.9-27.5)
Non-communicable disease	80	55.6 (47.3-63.5)	64	44.4 (36.5-52.7)
Maternal	1	100	0	0
External	19	90.5 (68.7-97.6)	2	9.5 (2.4-31.3)
Unspecified/ other	7	41.2 (20.9-64.9)	10	58.8 (35.1-79.1)
**Total**	176	64.2 (58.3-69.7)	98	35.8 (30.3-41.7)

1. Based on responses to questions on disability at the last time of asking before death, only including those participants with recorded cause of death

Figures

Figure 1. Flow chart of participation

For peer review only

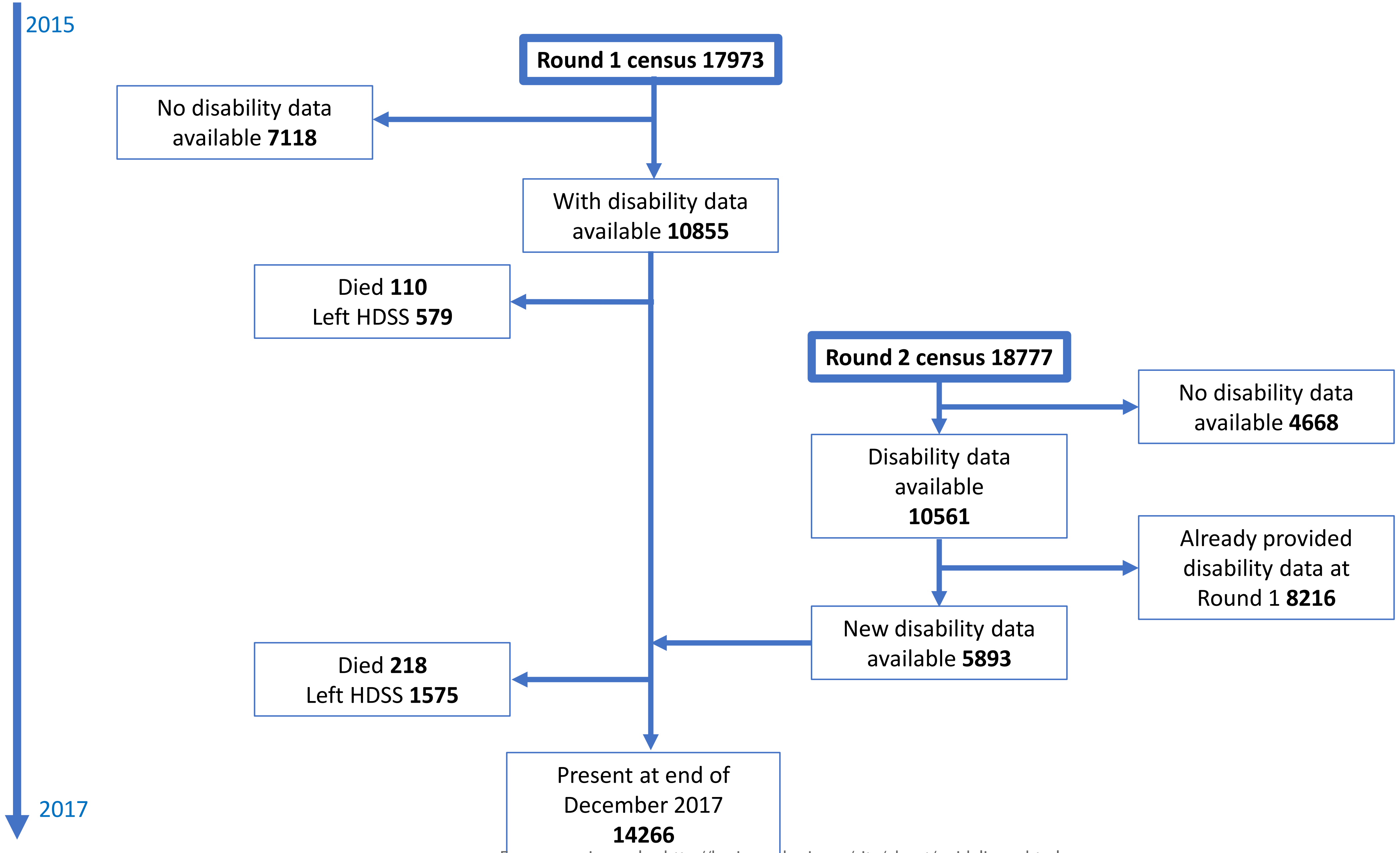
2015
Round 1 census 17973
No disability data available 7118
With disability data available 10855
Died 110
Left HDSS 579
Round 2 census 18777
No disability data available 4668
Disability data available 10561
Already provided disability data at Round 1 8216
New disability data available 5893
Died 218
Left HDSS 1575
Present at end of December 2017 14266
2017

Table S1. Age-specific all-cause mortality rates of men with and without disability data[1]

	With disability data			Without disability data		
**Age group**	**Number of deaths**	**Person-years**	**Rate per 1000 PY**	**Number of deaths**	**Person-years**	**Rate per 1000 PY**
**18-49**	56	10198	5.49 (4.23-7.14)	29	5502	5.27 (3.66-7.86)
**50-69**	56	2207	25.37 (19.52-32.97)	18	3623	36.23 (22.82-57.50)
**70+**	61	972	62.74 (48.81-80.63)	12	65	184.23 (104.63-324.40)

1. p value for heterogeneity between men with and without disability data = 0.94

Table S2. Poisson regression analysis of effect of graded levels of self-reported disability on all-cause mortality

	**Number of deaths**	**Person-years**	**Model 1: adjusted for age only[1]**		**Model 2: adjusted for age, sex, & occupation[2]**		**Model 3: adjusted for age, sex, occupation, BMI, hypertension, diabetes, & HIV status[3,4]**	
			RR (95% CI)	p- value[5]	RR (95% CI)	p- value[5]	RR (95% CI)	p- value[5]
**Any disability**								
**No difficulty**	48	13248	1	0.12	1	0.10	1	0.09
**Some difficulty**	82	7537	1.31 (0.87-1.99)		1.51 (0.99-2.29)		1.50 (0.99-2.28)	
**A lot of difficulty/ can't do at all**	50	1937	1.71 (1.02-2.86)		1.67 (0.98-2.85)		1.71 (1.01-2.90)	

1. Adjusted for age (continuous variable); excluding participants who were missing data on occupation, BMI, hypertension, diabetes, or HIV to allow comparison with Models 2 and 3
2. Adjusted for age (continuous variable), sex (male/female), and occupation (not working, manual work, farming/fishing, non-manual work); excluding participants who were missing data on BMI, hypertension, diabetes, or HIV to allow comparison with Models 1 and 3
3. Adjusted for age (continuous variable), sex (male/female), occupation (not working, manual work, farming/fishing, non-manual work), BMI (<18.5kg/m^2, 18.5-24.9kg/m^2, 25-29.9kg/m^2, 30+kg/m^2), hypertension (defined as a measured BP >140/90 or on antihypertensive medication), diabetes (defined as fasting blood sugar >7.0 or previous diagnosis of diabetes), and HIV status (defined as a positive HIV test or diagnosis ever)
4. Likelihood ratio test for heterogeneity in the association of disability and all-cause mortality by these factors: BMI p=0.08; hypertension p=0.67; diabetes p=0.41; HIV p=0.88
5. P value is a likelihood ratio test for difference

For peer review only

Table S3. Poisson regression analysis of effect of graded levels of self-reported disability on all-cause mortality by hypertension status (including unknown)[1,2]

	No hypertension			Hypertension			Unknown hypertension status		
	Deaths	Person-years	RR (95% CI)	Deaths	Person-years	RR (95% CI)	Deaths	Person-years	RR (95% CI)
**No disability**	48	14009	1	9	1286	1	27	7099	1
**Some difficulty**	59	6719	1.48 (0.99-2.20)	34	1790	1.72 (0.82-3.61)	30	2217	1.98 (1.14-3.41)
**A lot of difficulty / can't do at all**	30	1461	1.93 (1.16-3.20)	40	844	2.27 (1.07-4.81)	51	588	5.10 (2.94-8.83)

1. All models are controlled for age (continuous variable), sex, occupation (not working, manual work, farming/fishing, non-manual work)
2. p-value for heterogeneity = 0.03

For peer review only

STROBE Statement—checklist of items that should be included in reports of observational studies

	Item No	Recommendation	
**Title and abstract**	1	(*a*) Indicate the study's design with a commonly used term in the title or the abstract	Page 1
		(*b*) Provide in the abstract an informative and balanced summary of what was done and what was found	Page 2
**Introduction**			
Background/ rationale	2	Explain the scientific background and rationale for the investigation being reported	Page 4
Objectives	3	State specific objectives, including any prespecified hypotheses	Page 5
**Methods**			
Study design	4	Present key elements of study design early in the paper	Pages 5-6
Setting	5	Describe the setting, locations, and relevant dates, including periods of recruitment, exposure, follow-up, and data collection	Pages 5-6
Participants	6	(*a*) *Cohort study*—Give the eligibility criteria, and the sources and methods of selection of participants. Describe methods of follow-up<br>*Case-control study*—Give the eligibility criteria, and the sources and methods of case ascertainment and control selection. Give the rationale for the choice of cases and controls<br>*Cross-sectional study*—Give the eligibility criteria, and the sources and methods of selection of participants	Page 6
		(*b*) *Cohort study*—For matched studies, give matching criteria and number of exposed and unexposed<br>*Case-control study*—For matched studies, give matching criteria and the number of controls per case	
Variables	7	Clearly define all outcomes, exposures, predictors, potential confounders, and effect modifiers. Give diagnostic criteria, if applicable	Pages 6-7
Data sources/ measurement	8*	For each variable of interest, give sources of data and details of methods of assessment (measurement). Describe comparability of assessment methods if there is more than one group	Pages 5-6
Bias	9	Describe any efforts to address potential sources of bias	Pages 5-7
Study size	10	Explain how the study size was arrived at	n/a
Quantitative variables	11	Explain how quantitative variables were handled in the analyses. If applicable, describe which groupings were chosen and why	Pages 6-7
Statistical methods	12	(*a*) Describe all statistical methods, including those used to control for confounding	Page 7
		(*b*) Describe any methods used to examine subgroups and interactions	Page 7
		(*c*) Explain how missing data were addressed	Page 7
		(*d*) *Cohort study*—If applicable, explain how loss to follow-up was addressed<br>*Case-control study*—If applicable, explain how matching of cases and controls was addressed<br>*Cross-sectional study*—If applicable, describe analytical methods taking account of sampling strategy	Page 7
		(*e*) Describe any sensitivity analyses	Page 7

For peer review only

130-131

Continued on next page

For peer review only

For peer review only - http://bmjopen.bmj.com/site/about/guidelines.xhtml

**Results**			
Participants	13*	(a) Report numbers of individuals at each stage of study—eg numbers potentially eligible, examined for eligibility, confirmed eligible, included in the study, completing follow-up, and analysed	Figure 1
		(b) Give reasons for non-participation at each stage	Figure 1
		(c) Consider use of a flow diagram	Figure 1
Descriptive data	14*	(a) Give characteristics of study participants (eg demographic, clinical, social) and information on exposures and potential confounders	Table 1
		(b) Indicate number of participants with missing data for each variable of interest	Table 1
		(c) *Cohort study*—Summarise follow-up time (eg, average and total amount)	Page 8
Outcome data	15*	*Cohort study*—Report numbers of outcome events or summary measures over time	Page 8
		Case-control study—Report numbers in each exposure category, or summary measures of exposure	
		Cross-sectional study—Report numbers of outcome events or summary measures	
Main results	16	(*a*) Give unadjusted estimates and, if applicable, confounder-adjusted estimates and their precision (eg, 95% confidence interval). Make clear which confounders were adjusted for and why they were included	Table 2
		(*b*) Report category boundaries when continuous variables were categorized	
		(*c*) If relevant, consider translating estimates of relative risk into absolute risk for a meaningful time period	
Other analyses	17	Report other analyses done—eg analyses of subgroups and interactions, and sensitivity analyses	Supplementary tables
**Discussion**			
Key results	18	Summarise key results with reference to study objectives	Page 10
Limitations	19	Discuss limitations of the study, taking into account sources of potential bias or imprecision. Discuss both direction and magnitude of any potential bias	Page 11-12
Interpretation	20	Give a cautious overall interpretation of results considering objectives, limitations, multiplicity of analyses, results from similar studies, and other relevant evidence	Page 12
Generalisability	21	Discuss the generalisability (external validity) of the study results	Page 12
**Other information**			
Funding	22	Give the source of funding and the role of the funders for the present study and, if applicable, for the original study on which the present article is based	Page 14

*Give information separately for cases and controls in case-control studies and, if applicable, for exposed and unexposed groups in cohort and cross-sectional studies.

Note: An Explanation and Elaboration article discusses each checklist item and gives methodological background and published examples of transparent reporting. The STROBE checklist is best used in conjunction with this article (freely available on the Web sites of PLoS Medicine at http://www.plosmedicine.org/, Annals of Internal Medicine at http://www.annals.org/, and Epidemiology at http://www.epidem.com/). Information on the STROBE Initiative is available at www.strobe-statement.org.

For peer review only

For peer review only - http://bmjopen.bmj.com/site/about/guidelines.xhtml

BMJ Open

Self-reported disability in relation to mortality in rural Malawi: a longitudinal study of over 16000 adults

Journal:	*BMJ Open*
Manuscript ID	bmjopen-2019-034802.R1
Article Type:	Original research
Date Submitted by the Author:	16-Jan-2020
Complete List of Authors:	Prynn, Josephine; University College London,<br>Dube, Albert; Malawi Epidemiology and Intervention Research Unit<br>Mkandawire, Joseph; Malawi Epidemiology and Intervention Research Unit<br>Koole, Olivier ; London School of Hygiene and Tropical Medicine Faculty of Infectious and Tropical Diseases<br>Geis, Steffen; Malawi Epidemiology and Intervention Research Unit; London School of Hygiene and Tropical Medicine Faculty of Infectious and Tropical Diseases<br>Mwaiyeghele, Elenaus; Malawi Epidemiology and Intervention Research Unit<br>Mwiba, Oddie; Malawi Epidemiology and Intervention Research Unit<br>Price, Alison; London School of Hygiene and Tropical Medicine Faculty of Infectious and Tropical Diseases<br>Kachiwanda, Lackson; Malawi Epidemiology and Intervention Research Unit<br>Nyirenda, Moffat; London School of Hygiene and Tropical Medicine Faculty of Infectious and Tropical Diseases; MRC/UVRI Uganda Research Unit On AIDS<br>Kuper, Hannah; London School of Hygiene and Tropical Medicine, Faculty of Epidemiology and Population Health<br>Crampin, Amelia; London School of Hygiene and Tropical Medicine Faculty of Infectious and Tropical Diseases; Malawi Epidemiology and Intervention Research Unit
**Primary Subject Heading**:	Epidemiology
Secondary Subject Heading:	Global health, Public health
Keywords:	EPIDEMIOLOGY, Hypertension < CARDIOLOGY, General diabetes < DIABETES & ENDOCRINOLOGY, HIV & AIDS < INFECTIOUS DISEASES, PUBLIC HEALTH

SCHOLARONE™
Manuscripts

I, the Submitting Author has the right to grant and does grant on behalf of all authors of the Work (as defined in the below author licence), an exclusive licence and/or a non-exclusive licence for contributions from authors who are: i) UK Crown employees; ii) where BMJ has agreed a CC-BY licence shall apply, and/or iii) in accordance with the terms applicable for US Federal Government officers or employees acting as part of their official duties; on a worldwide, perpetual, irrevocable, royalty-free basis to BMJ Publishing Group Ltd ("BMJ") its licensees and where the relevant Journal is co-owned by BMJ to the co-owners of the Journal, to publish the Work in this journal and any other BMJ products and to exploit all rights, as set out in our licence.

The Submitting Author accepts and understands that any supply made under these terms is made by BMJ to the Submitting Author unless you are acting as an employee on behalf of your employer or a postgraduate student of an affiliated institution which is paying any applicable article publishing charge ("APC") for Open Access articles. Where the Submitting Author wishes to make the Work available on an Open Access basis (and intends to pay the relevant APC), the terms of reuse of such Open Access shall be governed by a Creative Commons licence – details of these licences and which Creative Commons licence will apply to this Work are set out in our licence referred to above.

Other than as permitted in any relevant BMJ Author's Self Archiving Policies, I confirm this Work has not been accepted for publication elsewhere, is not being considered for publication elsewhere and does not duplicate material already published. I confirm all authors consent to publication of this Work and authorise the granting of this licence.

For peer review only

Self-reported disability in relation to mortality in rural Malawi: a longitudinal study of over 16000 adults

Corresponding Author

Dr Josephine E Prynn, University College London, London UK; Malawi Epidemiology and Intervention Research Unit (MEIRU), Lilongwe, Malawi
Josephineprynn@gmail.com
+447746427488
187 Jamaica St, London E1 0PE

Co-authors

Mr Albert Dube, Malawi Epidemiology and Intervention Research Unit (MEIRU), Lilongwe, Malawi

Dr Joseph Mkandawire, Malawi Epidemiology and Intervention Research Unit (MEIRU), Lilongwe, Malawi

Dr Olivier Koole, London School of Hygiene and Tropical Medicine, London, UK

Dr Steffen Geis, Malawi Epidemiology and Intervention Research Unit (MEIRU), Lilongwe, Malawi; London School of Hygiene and Tropical Medicine, London, UK; Institute for Medical Microbiology and Hygiene, Philipps University of Marburg, Marburg, Germany

Elenaus Mwaiyeghele, Malawi Epidemiology and Intervention Research Unit (MEIRU), Lilongwe, Malawi

Mr Oddie Mwiba, Malawi Epidemiology and Intervention Research Unit (MEIRU), Lilongwe, Malawi

Dr Alison Price, London School of Hygiene and Tropical Medicine, London, UK

Mr Lackson Kachiwanda, Malawi Epidemiology and Intervention Research Unit (MEIRU), Lilongwe, Malawi

Prof Moffat Nyirenda, Malawi Epidemiology and Intervention Research Unit (MEIRU), Lilongwe, Malawi; London School of Hygiene and Tropical Medicine, London, UK; London School of Hygiene and Tropical Medicine, London, UK

Prof Hannah Kuper, London School of Hygiene and Tropical Medicine, London, UK

Prof Amelia C Crampin, Malawi Epidemiology and Intervention Research Unit (MEIRU), Lilongwe, Malawi**;** London School of Hygiene and Tropical Medicine, London, UK

Keywords or phrases
Disability; Epidemiology of chronic non communicable diseases; epidemiology; developing countries; mortality

Word count: 3121

For peer review only

For peer review only - http://bmjopen.bmj.com/site/about/guidelines.xhtml

Abstract

Objectives We investigated whether self-reported disability was associated with mortality in adults in rural Malawi.

Setting Karonga Health and Demographic Surveillance Site (HDSS), Northern Malawi

Participants All adults aged 18 and over residing in the HDSS were eligible to participate. During annual censuses in 2014 and 2015, participants were asked if they experienced difficulty in any of six functional domains and were classified as having disabilities if they reported "a lot of difficulty" or "can't do at all" in any domain. Mortality data was collected until December 31st, 2017. 16748 participants (10153 women and 6595 men) were followed up for a median of 29 months.

Primary and secondary outcome measures We used Poisson regression to examine the relationship between disability and all-cause mortality adjusting for confounders. We assessed whether this relationship altered in the context of obesity, hypertension, diabetes, or HIV. We also evaluated whether mortality from NCDs was higher among people who had reported disability, as determined by verbal autopsy.

Results: At baseline 7.6% reported a disability and the overall adult mortality rate was 9.4/1000 person-years. Adults reporting disability had an all-cause mortality rate 2.70 times higher than those without, and mortality rate from non-communicable disease 2.33 time higher than those without.

Conclusions Self-reported disability predicts mortality at all adult ages in rural Malawi. Interventions to improve access to healthcare and other services are needed.

For peer review only - http://bmjopen.bmj.com/site/about/guidelines.xhtml

Strengths and Limitations of This Study

- We were able to use high-quality mortality data collected through a well-functioning health and demographic surveillance survey in a country without complete vital registration
- The large sample size allowed us to examine the relationship between disability and mortality over a relatively short follow-up period
- This is the first study to examine whether disability as defined by the Washington Group questions is predictive of mortality, so contributes substantially to the existing literature on this topic
- A limitation of the study is that we have considerable missing data in chronic health states (BMI, hypertension, diabetes, HIV) which limits our understanding of how these are related to disability and mortality

Abbreviations

Body mass index (BMI)

Health and Demographic Surveillance Site (HDSS)

Non-communicable diseases (NCDs)

Sub-Saharan Africa (SSA)

Sustainable Development Goals (SDGs)

For peer review only

For peer review only - http://bmjopen.bmj.com/site/about/guidelines.xhtml

Introduction

Disability is a complex concept encompassing long-term physical, mental, intellectual or sensory impairments which, in interaction with personal and environmental factors, may limit people's participation in society on an equal basis with others.[1] The World Health Organisation (WHO) estimates that one billion people globally have a disability,[1] and the number is expected to rise further as the global population continues growing, life expectancy rises, and average age increases. People with disabilities are being "left behind" in terms of inclusion in education, employment, and social life,[1] and are at high risk of poverty.[2] Consequently, disability is recognised as an important development issue, and is explicitly referenced within five of the Sustainable Development Goals (SDGs).[3]

Disability is expected to be linked to increased mortality, through various pathways. Both disability and mortality are related to ageing and poverty, and so associations may arise through confounding.[1, 2] An underlying disease may also cause both disability and mortality, for instance diabetes can lead to impairments (e.g. visual impairment from diabetic retinopathy) and premature death. People with disabilities may find it more difficult to seek health-care due to a range of barriers,[1] thereby increasing their mortality risk. They may also have unhealthier behaviours, on average, regarding smoking, physical activity, and diet, increasing their vulnerability to obesity, non-communicable diseases (NCDs) and mortality.[4] There is also evidence that people with disabilities are more vulnerable to HIV and other infectious diseases, due to their marginalised position in society.[5] Certain impairments, such as mobility or cognitive impairments, can increase frailty, depression and functional difficulties, all linked to increased mortality.[6-8] Data are, however, lacking on the link between disability and mortality, particularly from low- and middle-income settings, where over 80% of people with disabilities reside.[9] The debate has raged as to how disability should be defined and measured, yet scales are needed to estimate the prevalence and impacts of disability, and to allow international data comparisons. Consensus is growing on the use of the Washington Group (WG) Short Set to collect Disability Statistics,[10] which will improve data comparability. The WG questions focus on difficulties in functioning in 6 different domains related to activities (e.g. walking) and participation (e.g. performing usual activities).

For peer review only - http://bmjopen.bmj.com/site/about/guidelines.xhtml

For peer review only

The objective of this study was to investigate the relationship between self-reported disability and mortality among adults in rural Malawi. A secondary objective was to assess whether the effect of disability on mortality varies with co-existence of obesity or a chronic disease (hypertension, diabetes, or HIV), and by cause of death.

Methods

Setting and data collection

The Karonga Health and Demographic Surveillance Site (HDSS) in Northern Malawi comprises a population of around 40,000 individuals, which is largely representative of the rural Malawian population in terms of age and sex structure.[11, 12] Census information is collected on the population annually, along with continuous reporting of births, deaths, and migration by community key informants. Verbal autopsy is done after every death using a semi-structured interview of a family member using an adaptation of a WHO instrument.[13] Two clinically trained reviewers independently assign cause of death based on this. In case of disagreement, a third reviewer arbitrates.

Since 2014, the WG short set questions on disability have been added to the annual census questionnaire, alongside the existing questions on demographic, health, and social indicators. The disability questions were only asked if the participant was physically seen by the fieldworker, although they could be answered through a proxy, after obtaining written consent or assent. Therefore, no disability data was collected from anyone away from home on the day of the census.

The questions, translated into the local language of Chitumbuka, are:

- Do you have difficulty seeing, even if wearing glasses?
- Do you have difficulty hearing, even if using a hearing aid?
- Do you have difficulty walking or climbing steps?
- Do you have difficulty remembering or concentrating?
- Do you have difficulty (with self-care such as) washing all over or dressing?

For peer review only - http://bmjopen.bmj.com/site/about/guidelines.xhtml

- Using your usual (customary) language, do you have difficulty communicating, for example understanding or being understood?

In this analysis, we used data on adults aged 18 and over from the first two consecutive census rounds to include the disability questions. The first was in 2014-2015 (Round 1), the second in 2015-2016 (Round 2). Baseline disability status was taken from the Round 1 census data where possible. For participants with no disability data from Round 1, data from Round 2 was used. Anyone who moved into the HDSS between Rounds 1 and 2, or turned 18 between Rounds 1 and 2, and answered disability questions at Round 2 was also included. Other socio-demographic information was taken from the same interview as the disability data. Follow-up was undertaken until 31st December 2017.

Data on body mass index (BMI), hypertension, and diabetes were gathered from a survey of all adults performed within the HDSS population in 2013-2015 on prevalence of major NCDs and their risk factors.[14] In this screening, height and weight were measured twice, and the mean was used to calculate BMI. Participants were asked if they had previously been diagnosed with hypertension or diabetes, and whether they were taking any regular medication. Resting blood pressure was measured three times with 5 minutes in between, and a mean of the second and third measures were used. Blood was taken for plasma glucose measurement after at least 8 hours fasting. This data was collected a mean of 1.2 years prior to the disability data (maximum 3.5 years).

Data on HIV status was collated from numerous sources including an HIV serosurvey in 2011 and the 2013-2015 NCD survey. Data on new HIV diagnoses was also collected from consenting participants at government clinics within the HDSS. Participants were categorised as HIV-positive if they had ever reported a diagnosis of HIV or had a positive antibody test. They were categorised as negative for 4 years after a negative HIV-test, after which time their status was categorised as missing in case of a new infection in the interim.

Variables

Age was grouped into categories of 18-34, 35-44, 45-54, 55-64, 65-69, 70-74, 75-79, and 80+ years; narrower age bands were chosen at higher ages as self-reported disability is strongly associated with older age. Education level was defined as no education, primary (including partially-completed), secondary (including partially-completed), and tertiary. Occupation was categorised into not working (including unemployed, unable to work, and retired), manual

For peer review only - http://bmjopen.bmj.com/site/about/guidelines.xhtml

work, farming or fishing, and non-manual work (including professional work). Participants were defined as in a union if they were married or co-habiting, and not if single, divorced, or widowed.

Categories of BMI were <18.5kg/m² (underweight), 18.5-24.9kg/m² (healthy weight), 25-29.9kg/m² (overweight), and 30+kg/m² (obese). Hypertension was defined as systolic blood pressure ≥140mmHg, diastolic blood pressure of ≥90mmHg, or use of antihypertensive medications. Diabetes was defined as fasting blood sugar ≥7.0mmol/L or a self-reported diagnosis of diabetes. Disability was defined as answering "a lot of difficulty" or "can't do at all" in any domain.

Cause of death among adults was broadly categorised into communicable disease, NCDs, maternal death, external (including injury and poisoning), and unspecifiable/other.

Statistical analysis

Prevalence of self-reported disability was calculated with 95% confidence intervals by socio-demographic and health characteristics. Poisson regression analysis was used to calculate adult mortality rate ratios, comparing people reporting "some difficulty" and "a lot of difficulty or can't do at all" with "no difficulty" in each disability domain. For these analyses, individuals contributed exposure time during their residence in the HDSS from the date the disability survey was completed, until the earliest of 31st December 2017, death, or out-migration. Returning and repeat migrants only contributed person-years while resident in the HDSS.

Age and sex were included *a priori* in the adjusted model, and baseline occupation, education level, and union status were sequentially added to the model to check for confounding. Any variable that altered the rate ratio more than 10% was kept in the adjusted model. Each of obesity, hypertension, diabetes and HIV status at baseline were also added to the models to check for confounding or effect modification. Cause-specific mortality was also calculated. We performed complete case analysis, so that any participants with missing data for any of the variables in the model were excluded. Sensitivity analyses, including an "unknown" category for BMI, hypertension, diabetes, and HIV were also performed.

All significance tests were likelihood-ratio tests.

Ethical approval

For peer review only - http://bmjopen.bmj.com/site/about/guidelines.xhtml

Ethical approval for the HDSS census rounds and NCD survey was granted by the National Health Sciences Research Committee (NHSRC) (protocol numbers #419 and #1072 respectively), and by the London School of Hygiene and Tropical Medicine (LSHTM) (protocol numbers #5081 and #6303 respectively).

Patient and public involvement

MEIRU works closely with the community in which this research was conducted. Regular meetings with senior community-members take place to ensure that study objectives align with the priorities of the community, and that the methodology and procedures are appropriate and acceptable. Research findings are disseminated similarly.

For peer review only

Results

Of 17973 adult (≥18 years) residents seen at Round 1, 10855 (60.4%) responded to questions on disability. A further 5893 adults not included at Round 1 (because they were under 18 years, not living in the area at Round 1, or missed) provided disability data at Round 2, giving 16748 participants with baseline disability data (**Figure 1**).

Those without disability data were more likely to be younger (22.3% of 18-39-year-olds compared to 10.8% of those aged 40+) and male (29.4% of men compared to 9.5% of women). Baseline characteristics of the census population compared to those included in the study are in **Table S1**. Participants with disability data had a median follow-up of 29 months (interquartile range 20-33 months). Migration out of the HDSS led to censoring of data in 1574 (9.5%) of participants. Individuals who migrated were more likely to be young and have higher educational attainment.

Table 1 shows that most participants were under age 35, and 76.1% of women and 62.5% of men were farmers or fishermen. The prevalence of obesity was higher in women than men (7.4% vs. 1.3%), whereas prevalence of hypertension, diabetes, and HIV were similar among men and women. Hypertension, diabetes, and HIV variables were not available for at least 30% of participants.

Prevalence of self-reported disability in one or more domain was 7.6% and strongly associated with increasing age (p test-for-trend <0.001). Adjusted for age, people with lower levels of education and those not working were more

For peer review only - http://bmjopen.bmj.com/site/about/guidelines.xhtml

likely to report disability, and prevalence was higher among participants with obesity, hypertension, or diabetes. People living with HIV reported a similar prevalence of disability to those who were HIV negative.

There were 328 deaths in 36019 person-years of follow-up. The adult mortality rate was 9.1/1000 person-years (95% CI 8.2-10.1) and higher among men (12.9/1000 person-years; 95%CI 11.1-15.0) than women (6.8/1000 person-years; 95% CI 5.8-8.0) including when controlling for age (rate ratio 1.75; 95% CI 1.44-2.14). Sensitivity analyses comparing all-cause mortality rates in men without disability data to those with disability data suggested no material difference stratified by age (**Table S2**).

Table 2 shows that compared with adults reporting "no difficulty" in any disability domain, those reporting "some difficulty", and "a lot of difficulty or can't do at all" had greater mortality (RR 1.39; 95% CI 1.03-1.88 and RR 2.70; 95% CI 1.91-3.82, respectively). Except for difficulty seeing and hearing, self-reported disability in any of the domains was strongly associated with increased mortality when adjusted for age, and the association remained when also adjusted for sex and occupation. For every domain, reporting disability ("a lot of difficulty" or "can't do at all") was associated with a higher mortality than answering "some difficulty". Disability in increasing numbers of domains was strongly associated with mortality. The relationship between any self-reported disability and all-cause mortality did not vary by sex or age group (**Table 3**); and none of BMI, hypertension, diabetes, or HIV were confounders or effect modifiers of the relationship (**Table S3**). Sensitivity analysis including a "missing" category for hypertension did show heterogeneity by hypertension status (p=0.03): the relationship between disability and mortality was stronger among those with unknown hypertension status (**Table S4**). Including a "missing" category for BMI, diabetes, and HIV did not show any heterogeneity.

Table 4 shows that mortality from non-communicable diseases was higher among people living with disability than those without disability. No difference was seen for mortality from HIV or other communicable diseases.

Discussion

For peer review only - http://bmjopen.bmj.com/site/about/guidelines.xhtml

Our findings show that self-reported disability in adults is strongly associated with increased all-cause mortality in rural Malawi. Furthermore, a stepwise increase in risk was observed with increasing levels of difficulties in functioning and numbers of domains affected. Individuals reporting difficulty in walking and self-care were particularly at risk. The co-existence of obesity or chronic disease did not materially alter estimates of effect, and the magnitude of the association was consistent across age- and sex- groups. People dying from NCDs were more likely to have been living with disability before death than those dying from HIV.

Our findings are similar to those observed elsewhere in sub-Saharan Africa (SSA). A study in Tanzanian adults aged 70+ also found a stepwise increase in mortality with increasing disability severity.[15] In South African adults aged 50+, severe functional disability was associated with a mortality rate ratio of 1.92 in women and 2.80 in men, compared to those with least functional disability.[16] In Malawi, Payne et al described an increasing mortality risk with increasingly severe disability among adults aged 45 and over, compared with those without disability.[17] None of these studies used the WG questions, however.

The association between disability and mortality has also been observed in high-income countries (HIC).[18-21] This is seen even after adjusting for a wide range of comorbidities,[22-24] suggesting that the association between disability and mortality is not solely due to conditions predisposing to both disability and death. Nonetheless, contexts differ widely between high-resource settings and rural Malawi hence these findings may not be generalizable to our setting. In HIC, improved access to healthcare means that many more people live with treated chronic disease over many years, as opposed to in a low -income country (LIC) like Malawi, where people may be more likely to die early from disabling disease.[14, 25] Even access to safe water and sanitation services can be profoundly affected by disability in LIC including Malawi,[26, 27] which is an important risk factor for disease.

We observed a higher prevalence of disability in women than men at all ages, yet mortality rates were consistently higher in men than women across all adult age groups, irrespective of disability. This apparent "male-female health survival paradox" has been seen in a wide variety of settings, using a range of disability definitions.[15, 16, 23]

The population in Malawi is ageing, as in much of SSA.[28] As such, the very strong positive correlation between increasing age and prevalence of self-reported disability portends a growing prevalence of disability in the

For peer review only - http://bmjopen.bmj.com/site/about/guidelines.xhtml

population in coming decades. The extent to which increasing life expectancy leads to an increased number of years lived with disability, or whether onset of disability will be delayed with a healthier life-course leading to increased life expectancy, is not yet understood.[29]

A major strength of our study is the available high-quality mortality data from a long-established HDSS, when vital registration is very limited in Malawi.[30] Moreover, the large sample size and low loss to follow-up allows meaningful mortality analyses despite a relatively short follow-up time. Disability is not a static state: people move considerably between disability states over time.[17, 31] Despite this, a one-off self-report of disability put individuals at a much-increased risk of death in the following 2 years. As further disability and mortality data is collected with time, we will be able to examine mortality over a longer follow-up time and consider self-reported disability as a time-varying exposure in future analyses. Our study adds considerably to the very limited literature examining mortality with disability in this context and is the first to examine whether disability as defined by the WG questions is predictive of mortality.

Our study has several limitations. The WG questions do not capture a comprehensive picture of disability, for example common impairments such as affective disorders and chronic pain are not included. Furthermore, the use of self-report relies on participants having comparable interpretations of what constitutes "some difficulty" versus "a lot of difficulty". Self-report also requires people to be able to understand the questions and communicate their response, so there is a risk that some participants would have been unable to self-identify. However, by allowing proxy responses where necessary, this will have been partially mitigated. We have limited information on underlying health conditions in the population and our data on hypertension, diabetes, BMI, and HIV were not measured concurrently with disability. If participants were to change category in the intervening time misclassification could occur, but as these are all, in the main, long-term health states, it is unlikely that this will have affected a substantial number of participants. We had considerable missing data on hypertension, diabetes, and HIV status, and our data may have been more likely to capture positive cases than negative. For hypertension and diabetes, the data was missing mainly on younger, working men, who are likely to have lower prevalence than the general population.[30] For HIV status some data is from new diagnoses made at health centres, where people may present if they feel they are at risk of being HIV positive. Participants with missing data on hypertension demonstrated a stronger relationship between disability and mortality than those with known hypertension or

For peer review only - http://bmjopen.bmj.com/site/about/guidelines.xhtml

known normotension, suggesting that the hypertension data is not missing at random. This limits our understanding of the relationship of these conditions with disability and mortality, and more research is needed to see whether our findings are replicated in both this and other settings. Our pragmatic approach to only collect disability reports on individuals found at home during our annual census means that the lower reports of disability and increased mortality in men compared to women seen in all age groups could be suspected to be due to a "healthy worker" effect as more men than women work far from the household in paid employment. However, we found that particularly among younger men, the mortality rate was very similar among those included in this analysis and those excluded.

For peer review only

Conclusions

Disability is commonly reported and likely to increase in Malawi in coming decades as the population ages and NCDs become increasingly prevalent. Self-reported disability is associated with significantly increased mortality, which seems to be driven particularly by an increased mortality rate from non-communicable diseases. While more research is urgently needed to help understand the mechanism behind this relationship, this needs to be concurrent with active interventions to improve access to healthcare and other services, particularly for people with disabilities, if the commitment to "leave no-one behind"[32] in the SDGs is to be realised.

References

1. World Health Organisation, *World Report on Disability*. 2011.
2. Banks, L.M., H. Kuper, and S. Polack, *Poverty and disability in low- and middle-income countries: A systematic review.* PLOS ONE, 2017. **12**(12): p. e0189996.
3. United Nations. *Sustainable Development Goals (SDGs) and Disability*. 16th April 2019]; Available from: https://www.un.org/development/desa/disabilities/about-us/sustainable-development-goals-sdgs-and-disability.html.
4. Hashemi, G., M. Wickenden, and H. Kuper, *SDGs, Inclusive Health and the path to Universal Health Coverage.* Disability and the Global South, 2017. **4**(1): p. 23.
5. De Beaudrap, P., M. Mac-Seing, and E. Pasquier, *Disability and HIV: a systematic review and a meta-analysis of the risk of HIV infection among adults with disabilities in Sub-Saharan Africa.* AIDS Care, 2014: p. 1-10.

For peer review only - http://bmjopen.bmj.com/site/about/guidelines.xhtml

6. Liljas, A.E.M., et al., *Self-reported vision impairment and incident prefrailty and frailty in English community-dwelling older adults: findings from a 4-year follow-up study.* J Epidemiol Community Health, 2017. **71**(11): p. 1053-1058.
7. Ribeiro, M.V., et al., *Association between visual impairment and depression in the elderly: a systematic review.* Arq Bras Oftalmol, 2015. **78**(3): p. 197-201.
8. Cimarolli, V.R. and D.S. Jopp, *Sensory impairments and their associations with functional disability in a sample of the oldest-old.* Qual Life Res, 2014. **23**(7): p. 1977-84.
9. World Health Organisation, *Concept Note: World Report on Disability and Rehabilitation*. 2007.
10. *Washington Group on Disability Statistics* Available from: http://www.washingtongroup-disability.com/.
11. Malawi National Statistical Office, *Population and Housing Census Results* 2008.
12. Dube, A., et al., *Profile: The Karonga Health and Demographic Surveillance System.* International Journal of Epidemiology, 2012. **41**(3): p. 676-685.
13. World Health Organisation. *Verbal autopsy standards: ascertaining and attributing causes of death*. 2018 14th July 2019]; Available from: https://www.who.int/healthinfo/statistics/verbalautopsystandards/en/.
14. Price, A.J., et al., *Prevalence of obesity, hypertension, and diabetes, and cascade of care in sub-Saharan Africa: a cross-sectional, population-based study in rural and urban Malawi.* The Lancet Diabetes & Endocrinology, 2018. **6**(3): p. 208-222.
15. Gray, W.K., et al., *Rates and predictors of three-year mortality in older people in rural Tanzania.* Archives of Gerontology and Geriatrics, 2016. **62**(Supplement C): p. 36-42.
16. Gómez-Olivé, F.X., et al., *Social conditions and disability related to the mortality of older people in rural South Africa.* International Journal of Epidemiology, 2014. **43**(5): p. 1531-1541.
17. Payne, C., J. Mkandawire, and H.-P. Kohler, *Disability Transitions and Health Expectancies among Adults 45 Years and Older in Malawi: A Cohort-Based Model.* PLoS Med, 2013. **10**(5): p. e100435.
18. Park, J.M., et al., *Disparities in mortality by disability: an 11-year follow-up study of 1 million individuals.* International Journal of Public Health, 2017. **62**(9): p. 989-996.
19. Björkenstam, C., et al., *Diagnosis-specific disability pension and risk of all-cause and cause-specific mortality – a cohort study of 4.9 million inhabitants in Sweden.* BMC Public Health, 2014. **14**(1): p. 1247.
20. Karlsson, N.E., et al., *Mortality in relation to disability pension: Findings from a 12-year prospective population-based cohort study in Sweden.* Scandinavian Journal of Public Health, 2007. **35**: p. 341-347.
21. Forman-Hoffman, V.L., et al., *Disability status, mortality, and leading causes of death in the United States community population.* Med Care, 2015. **53**(4): p. 346-54.
22. Hennessy, S., et al., *Disability Stage is an Independent Risk Factor for Mortality in Medicare Beneficiaries Aged 65 Years and Older.* PM&R, 2015. **7**(12): p. 1215-1225.
23. Pongiglione, B., et al., *Disability and all-cause mortality in the older population: evidence from the English Longitudinal Study of Ageing.* European Journal of Epidemiology, 2016. **31**(8): p. 735-746.
24. Majer, I.M., et al., *Mortality risk associated with disability: a population-based record linkage study.* Am J Public Health, 2011. **101**(12): p. e9-15.
25. Dewhurst, F., et al., *The prevalence of disability in older people in Hai, Tanzania.* Age Ageing, 2012. **41**(4): p. 517-23.
26. Groce, N., et al., *Water and sanitation issues for persons with disabilities in low- and middle-income countries: a literature review and discussion of implications for global health and international development.* Journal of Water and Health, 2011. **9**(4): p. 617.
27. White, S., et al., *A qualitative study of barriers to accessing water, sanitation and hygiene for disabled people in Malawi.* PLOS ONE, 2016. **11**(5).
28. Division, U.N.D.P., *World Population Prospects: The 2017 Revision*. 2017.
29. Beard, J.R., et al., *The World report on ageing and health: a policy framework for healthy ageing.* Lancet, 2016. **387**: p. 2145-54.
30. Crampin, A.C., et al., *Hypertension and diabetes in Africa: design and implementation of a large population-based study of burden and risk factors in rural and urban Malawi.* Emerg Themes Epidemiol, 2016. **13**: p. 3.
31. Prynn, J.E., et al., *Self-reported disability in rural Malawi: prevalence, incidence, and relationship to chronic disease [version 1; peer review: awaiting peer review].* Wellcome Open Research, 2019. **4**(90).
32. United Nations. *The Sustainable Development Goals Report*. 2016 16th August 2018]; Available from: https://unstats.un.org/sdgs/report/2016/leaving-no-one-behind.

For peer review only - http://bmjopen.bmj.com/site/about/guidelines.xhtml

Footnotes

Author Contributions

Conceptualisation: OK, AP, HK, ACC

Funding acquisition: MN, ACC

Design: AD, SG, AP

Project administration: AD, JM, EM, OM

Supervision of data collection: AD. OK, SG, EM, AP, LK

Data curation: OM

Analysis: JEP, ACC

Original draft preparation: JEP, HK, ACC

Review and editing of manuscript: JEP, AD, JM, OK, SG, EM, OM, AP, LK, MN, HK, ACC

Funding statement

This work was supported by the Wellcome Trust, grant number #098610

Competing interests

There were no competing interests for any author

Data Sharing Agreement

Data from the NCD survey is available from LSHTM Data Compass: Malawi Epidemiology and Intervention Research Unit Non-Communicable Disease Survey data, 2013–2017. https://doi.org/10.17037/DATA.0000096136. Data are available under the terms of the Creative Commons Attribution 3.0 International license (CC-BY 3.0).

For peer review only - http://bmjopen.bmj.com/site/about/guidelines.xhtml

Summary demographic datasets are publicly available through the INDEPTH iShare platform.

Longitudinal data (demographic surveillance episodes and linked rounds of disability questionnaires) cannot be sufficiently de-identified for public availability. Application may be made for access through the MEIRU director (mia.crampin@lshtm.ac.uk) or data scientist Chifundo Kanjala (chifundo.kanjala@lshtm.ac.uk). Those wishing to access the data will need to provide a brief proposal for what the data will be used for as a condition of access.

For peer review only

For peer review only - http://bmjopen.bmj.com/site/about/guidelines.xhtml

Table 1. Baseline characteristics of participants with baseline disability data and proportion with self-reported disability[1]

	Women			Men			Total		
	**n**	**Number reporting disability (%)**	**p-value[2]**	**n**	**Number reporting disability (%)**	**p-value[2]**	**n**	**Number reporting disability (%)**	**p-value[2]**
**Overall**	10153	857 (8.4)	-	6595	420 (6.4)	-	16748	1277 (7.6)	-
**Age group**									
18-34	5503	167 (3.0)		3378	73 (2.2)		8881	240 (2.7)	
35-44	1914	103 (5.4)		1339	48 (3.6)		3253	151 (4.6)	
45-54	1100	122 (11.1)		786	55 (7.0)		1886	177 (9.4)	
55-64	773	116 (15.0)	<0.001*	486	64 (13.2)	<0.001*	1259	180 (14.3)	<0.001*
65-69	258	54 (20.9)		175	25 (14.3)		433	79 (18.2)	
70-74	212	75 (35.4)		133	38 (28.6)		345	113 (32.8)	
75-79	189	94 (49.7)		138	45 (32.6)		327	139 (42.5)	
80+	204	126 (61.8)		160	72 (45.0)		364	198 (54.4)	
**Education level[3]**									
None	378	117 (31.0)		98	27 (27.6)		476	144 (30.3)	
Some primary/completed primary	6603	632 (9.6)	0.002*	3284	269 (8.2)	0.06*	9887	901 (9.1)	<0.001*
Some secondary/completed secondary	2633	82 (3.1)		2707	99 (3.7)		5340	181 (3.4)	
Tertiary	469	15 (3.2)		467	24 (5.1)		936	39 (4.2)	
**Occupation[4]**									
Not working	1013	202 (19.9)		985	106 (10.8)		1998	308 (15.4)	
Manual	143	5 (3.5)		745	28 (3.8)		888	33 (3.7)	
Farmer/fisherman	7637	567 (7.4)	<0.001	4092	256 (6.3)	<0.001	11729	823 (7.0)	<0.001
Non-manual/ business/ professional	1249	62 (5.0)		724	26 (3.6)		1973	88 (4.5)	
**Union status[5]**									
Not in a union	3381	479 (14.2)	<0.001	1933	97 (5.0)	0.001	5314	576 (10.8)	<0.001
In a union	6768	378 (5.6)		4659	323 (6.9)		11427	701 (6.1)	
**BMI (kg/m²)[6]**									

For peer review only - http://bmjopen.bmj.com/site/about/guidelines.xhtml

<18.5	669	61 (9.1)	0.005	601	57 (9.5)	0.38	1270	118 (9.3)	<0.001
18.5-24.9	6074	423 (7.0)		4847	263 (5.4)		10921	686 (16.3)	
25-29.9	1869	176 (9.4)		497	50 (10.1)		2366	226 (9.6)	
30+	689	90 (13.1)		80	10 (12.5)		769	100 (13.0)	
**Hypertension[7]**									
No hypertension	6069	416 (6.9)	0.69	3599	200 (5.6)	0.05	9668	616 (6.4)	0.05
Hypertension	1011	242 (23.9)		633	110 (17.4)		1644	352 (21.4)	
**Diabetes[8]**									
No diabetes	6189	535 (8.6)	0.22	3582	259 (7.2)	0.007	9771	794 (8.1)	0.008
Diabetes	109	25 (22.9)		69	19 (27.5)		178	44 (24.7)	
**HIV status[9]**									
Negative	6128	490 (8.0)	0.69	3070	218 (7.1)	0.60	9198	708 (7.7)	0.90
Positive	907	70 (7.7)		443	38 (8.6)		1350	108 (8.0)	
**Number of chronic diseases[10,11]**									
0	4243	261 (6.2)	0.77*	2054	102 (5.0)	0.002*	6297	363 (5.8)	0.02*
1	1166	170 (14.6)		620	91 (14.7)		1786	261 (14.6)	
2	100	19 (19.0)		69	14 (20.3)		169	33 (19.5)	
3	5	1 (20.0)		2	1 (50.0)		7	2 (28.6)	

1. Defined as answering “a lot of difficulty” or “can’t do at all” to at least one disability domain
2. p-value for difference using likelihood ratio test, values marked as * (age, education level, number of chronic diseases) p-value for trend. For all variables other than age group this is controlled for age (as a continuous variable)
3. Education level: No data on 70 women and 39 men
4. Occupation: No data on 111 women and 49 men
5. Union status: No data on 4 women and 3 men
6. BMI: No data on 872 women and 570 men
7. Hypertension: No data on 3073 women and 2363 men
8. Diabetes: No data on 3855 women and 2944 men
9. HIV status: No data on 3118 women and 3082 men
10. Chronic diseases included in this variable: hypertension, diabetes, HIV
11. Number of chronic diseases variable: No data on 4639 women and 3850 men (only participants with data on all 3 chronic diseases included)

For peer review only

Table 2. Poisson regression analysis of effect of graded levels of self-reported disability on all-cause adult mortality

	Number of deaths	Person-years	Model 1: adjusted for age only[1]		Model 2: adjusted for age, sex, & occupation[2]	
			RR (95% CI)	p- value[3]	RR (95% CI)	p-value[3]
**Difficulty seeing**						
No difficulty	165	28106	1	0.38		0.85
Some difficulty	107	6316	1.02 (0.79-1.34)		1.08 (0.82-1.41)	
A lot of difficulty/ can't do at all	49	1255	1.20 (0.84-1.73)		1.01 (0.7-1.46)	
**Difficulty hearing**						
No difficulty	296	33922	1	0.05		0.19
Some difficulty	39	1480	1.20 (0.85-1.71)		1.11 (0.78-1.58)	
A lot of difficulty/ can't do at all	13	275	1.79 (1.01-3.15)		1.51 (0.85-2.68)	
**Difficulty walking**						
No difficulty	157	30222	1	<0.001		<0.001
Some difficulty	79	4175	1.39 (1.04-1.88)		1.57 (1.15-2.13)	
A lot of difficulty/ can't do at all	85	1278	3.40 (2.48-4.66)		3.20 (2.27-4.51)	
**Difficulty remembering**						
No difficulty	204	29608	1	<0.001	1	<0.001
Some difficulty	79	5384	1.06 (0.81-1.39)		1.17 (0.89-1.54)	
A lot of difficulty/ can't do at all	37	650	2.67 (1.84-3.85)		2.55 (1.74-3.73)	
**Difficulty communicating**						
No difficulty	287	35235	1	<0.001	1	<0.001
Some difficulty	23	332	3.11 (2.01-4.8)		2.26 (1.44-3.53)	
A lot of difficulty/ can't do at all	10	82	8.81 (4.67-16.59)		5.33 (2.78-10.21)	

For peer review only

**Difficulty with self-care**						
No difficulty	234	33850	1		1	
Some difficulty	47	1306	1.84 (1.33-2.56)	<0.001	1.74 (1.24-2.45)	<0.001
A lot of difficulty/ can't do at all	38	503	3.75 (2.62-5.38)		3.14 (2.15-4.6)	
**Any disability**						
No difficulty	84	22197	1		1	
Some difficulty	122	10645	1.39 (1.03-1.88)	<0.001	1.61 (1.18-2.19)	<0.001
A lot of difficulty/ can't do at all	115	2839	2.70 (1.91-3.82)		2.65 (1.84-3.8)	
**Disability in multiple domains**						
No disability	206	32848	1		1	
Disability in 1 domain	51	2018	1.60 (1.15-2.21)		1.51 (1.08-2.11)	
Disability in 2 domains	31	543	2.39 (1.59-3.59)	<0.001	2.07 (1.35-3.17)	<0.001
Disability in 3 domains	19	120	3.71 (2.26-6.09)		3.06 (1.83-5.14)	
Disability in 4+ domains	14	79	5.64 (3.19-9.96)		4.33 (2.39-7.88)	

1. Controlled for age (continuous variable)
2. Controlled for age (continuous variable), sex, and occupation (not working, manual work, farming/fishing, non-manual work)
3. Likelihood ratio test for difference

For peer review only

For peer review only - http://bmjopen.bmj.com/site/about/guidelines.xhtml

Table 3. Poisson regression of disability[1] on all-cause adult mortality stratified by age group and sex[1]

Age group		Women					Men				
		No. of deaths	PY[3]	Mortality rate per 1000 PY (95% CI)	RR[4] (95%CI)	p-value	No. of deaths	PY[4]	Mortality rate per 1000 PY (95% CI)	RR[3] (95% CI)	p-value
**18-49**	No disability	40	16741	2.39 (1.75-3.26)	1	0.04	51	9870	5.17 (3.93-6.80)	1	0.10
	Disability	6	739	8.12 (3.65-18.09)	2.76 (1.16-6.58)		5	324	15.43 (6.42-37.06)	2.37 (0.94-5.97)	
**50-69**	No disability	25	3169	7.89 (5.33-11.67)	1	0.002	44	1952	22.54 (16.78-30.29)	1	0.06
	Disability	14	566	24.74 (14.65-41.77)	3.00 (1.55-5.79)		12	255	46.98 (26.68-82.73)	1.93 (1.02-3.65)	
**70+**	No disability	18	749	24.04 (15.15-38.15)	1	<0.001	29	636	45.62 (31.70-65.64)	1	0.009
	Disability	52	673	77.30 (58.90-101.45)	2.76 (1.61-4.75)		32	337	95.07 (67.23-134.44)	1.98 (1.19-3.30)	
**All**	No disability	83	20659	4.02 (3.24-4.98)	1	<0.001	124	12458	9.95 (8.35-11.87)	1	0.001
	Disability	72	1977	36.42 (28.91-45.88)	2.89 (1.20-4.19)		49	916	53.49 (40.42-70.77)	1.87 (1.30-2.71)	

1. Disability is defined as reporting “a lot of difficulty” or “can’t do at all” in any of the functional domains
2. p-value for heterogeneity between age-sex groups = 0.94
3. Person-years
4. Rate ratio, adjusted for age (as a continuous variable)

For peer review only - http://bmjopen.bmj.com/site/about/guidelines.xhtml

For peer review only

Table 4. Cause-specific mortality rate ratio among those living with disability prior to death compared to those not living with disability

Cause of death[1]	Number who died		Mortality rate ratio[2]	p-value
	Disability	No disability		
**Communicable disease (excluding HIV)**	17	12	1.42 (0.62-3.26)	0.41
**HIV**	52	10	1.80 (0.89-3.65)	0.10
**Non-communicable disease**	80	64	2.33 (1.60-3.38)	<0.01
**Maternal**	1	0	-	-
**External**	19	2	1.22 (0.32-4.65)	0.77
**Unspecified/other**	7	10	3.92 (1.30-11.81)	0.02

1. **Cause of death** attributed by verbal autopsy
2. **Adjusted for age** (as a continuous variable)

For peer review only

Figures

Figure 1. Flow chart of participation

For peer review only - http://bmjopen.bmj.com/site/about/guidelines.xhtml

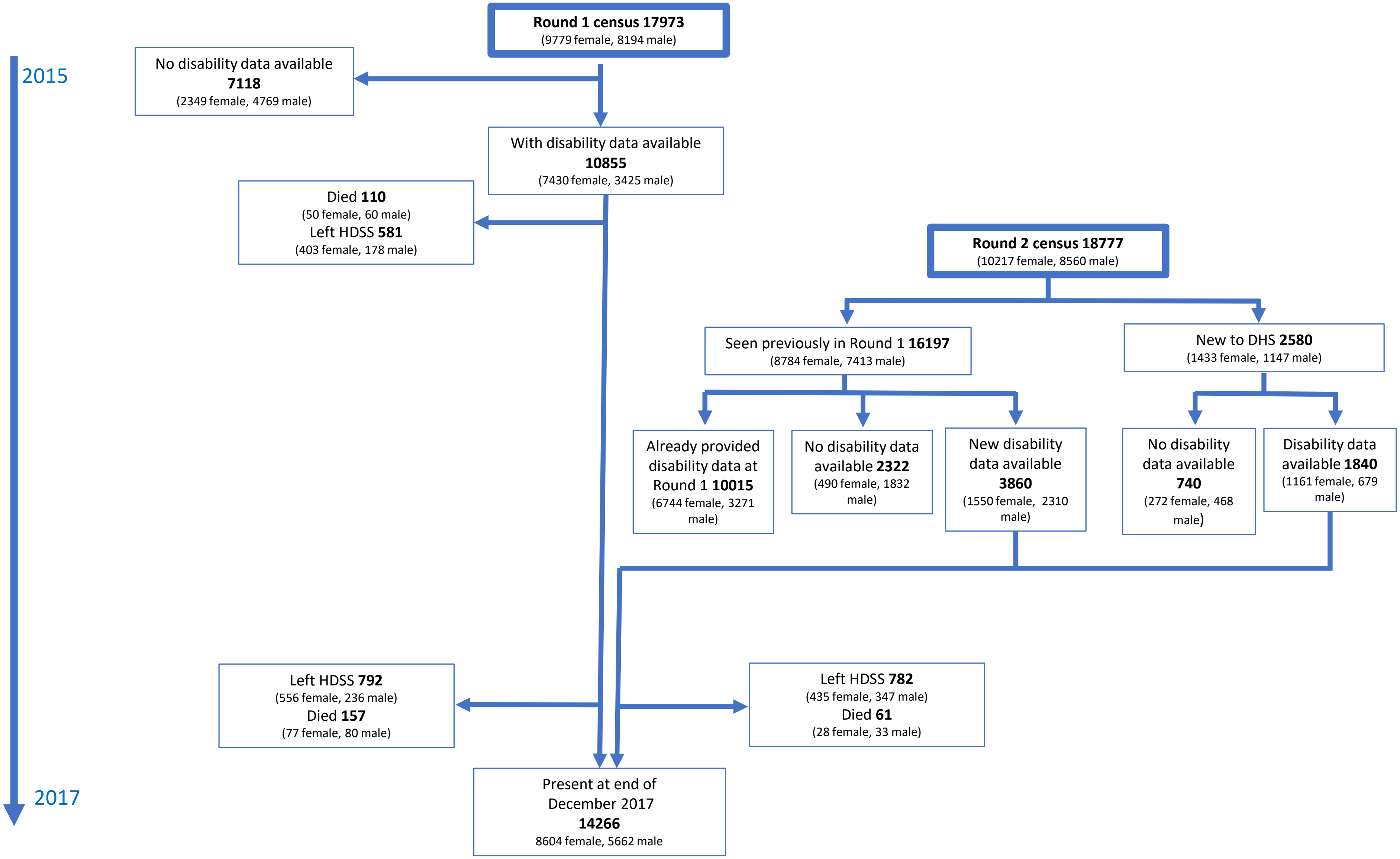
2015
2017
Round 1 census 17973
(9779 female, 8194 male)
No disability data available
7118
(2349 female, 4769 male)
With disability data available
10855
(7430 female, 3425 male)
Died 110
(50 female, 60 male)
Left HDSS 581
(403 female, 178 male)
Round 2 census 18777
(10217 female, 8560 male)
Seen previously in Round 1 16197
(8784 female, 7413 male)
New to DHS 2580
(1433 female, 1147 male)
Already provided disability data at Round 1 10015
(6744 female, 3271 male)
No disability data available 2322
(490 female, 1832 male)
New disability data available
3860
(1550 female, 2310 male)
No disability data available
740
(272 female, 468 male)
Disability data available 1840
(1161 female, 679 male)
Left HDSS 792
(556 female, 236 male)
Died 157
(77 female, 80 male)
Left HDSS 782
(435 female, 347 male)
Died 61
(28 female, 33 male)
Present at end of December 2017
14266
8604 female, 5662 male

Table S1. Baseline characteristics of census participants and study participants at Round 1

	Census			Study[1]		
	Women	Men	Total	Women	Men	Total
	n=9779	n=8194	n=17973	n=7430	n=3425	n=10855
**Age group**						
**18-34**	5142 (52.6)	4472 (54.6)	9614 (53.5)	3813 (51.3)	1544 (45.1)	5357 (49.4)
**35-44**	1874 (19.2)	1598 (19.5)	3472 (19.3)	1476 (19.9)	711 (20.8)	2187 (20.1)
**45-54**	1105 (11.3)	934 (11.4)	2039 (11.3)	814 (11.0)	443 (12.9)	1257 (11.6)
**55-64**	781 (8.0)	553 (6.7)	1334 (7.4)	606 (8.2)	306 (8.9)	912 (8.4)
**65-69**	280 (2.9)	192 (2.3)	472 (2.6)	213 (2.9)	105 (3.1)	318 (2.9)
**70-74**	197 (2.0)	142 (1.7)	339 (1.9)	173 (2.3)	101 (2.9)	274 (2.5)
**75-79**	200 (2.0)	154 (1.9)	354 (2.0)	164 (2.2)	96 (2.8)	260 (2.4)
**80+**	200 (2.0)	149 (1.8)	349 (1.9)	171 (2.3)	119 (3.5)	290 (2.7)
**Missing**	0	0	0	0	0	0
**Education level**						
**None**	379 (3.9)	104 (1.3)	483 (2.7)	296 (4.0)	69 (2.0)	365 (3.4)
**Some primary/completed primary**	6323 (65.1)	3961 (48.6)	10284 (57.6)	4975 (67.4)	1834 (53.7)	6809 (63.1)
**Some secondary/completed secondary**	2529 (26.1)	3442 (42.2)	5971 (33.4)	1757 (23.8)	1264 (37.0)	3021 (28.0)
**Tertiary**	477 (4.9)	649 (8.0)	1126 (6.3)	351 (4.8)	247 (7.2)	598 (5.5)
**Missing**	71	38	109	51	11	62
**Occupation**						
**not working**	1156 (12)	1497 (18.3)	2653 (14.9)	621 (8.4)	392 (11.5)	1013 (9.4)
**manual**	130 (1.3)	1086 (13.3)	1216 (6.8)	89 (1.2)	386 (11.3)	475 (4.4)
**farmer/fisherman**	7190 (74.3)	4639 (56.8)	11829 (66.3)	5716 (77.7)	2326 (68.1)	8042 (74.7)

For peer review only

**non-manual/ business/ professional**	1196 (12.4)	939 (11.5)	2135 (12)	928 (12.6)	310 (9.1)	1238 (11.5)
**Missing**	107	33	140	76	11	87
**Union status**						
**Not in a union**	3380 (34.6)	2635 (32.2)	6015 (33.5)	2339 (31.5)	869 (25.4)	3208 (29.6)
**In a union**	6391 (65.4)	5547 (67.8)	11938 (66.5)	5087 (68.5)	2553 (74.6)	7640 (70.4)
**Missing**	8	12	20	4	3	7
**BMI**						
**<18.5**	624 (7.2)	647 (9.6)	1271 (8.2)	482 (7.1)	298 (10.0)	780 (8.0)
**18.5-24.9**	5667 (65.0)	5434 (80.9)	11101 (71.9)	4344 (64.4)	2373 (79.8)	6717 (69.1)
**25-29.9**	1774 (20.3)	554 (8.2)	2328 (15.1)	1401 (20.8)	269 (9.0)	1670 (17.2)
**30+**	660 (7.6)	83 (1.2)	743 (4.8)	520 (7.7)	35 (1.2)	555 (5.7)
**Missing**	1054	1476	2530	683	450	1133
**Hypertension**						
**No hypertension**	6286 (85.8)	4639 (86.3)	10925 (86.0)	4871 (85.3)	2026 (82.4)	6897 (84.4)
**Hypertension**	1041 (14.2)	737 (13.7)	1778 (14.0)	837 (14.7)	434 (17.6)	1271 (15.6)
**Missing**	2452	2818	5270	1722	965	2687
**Diabetes**						
**No diabetes**	6400 (98.2)	4534 (98.2)	10934 (98.2)	4988 (98.3)	2057 (97.5)	7045 (98.1)
**Diabetes**	117 (1.8)	81 (1.8)	198 (1.8)	87 (1.7)	53 (2.5)	140 (1.9)
**Missing**	3262	3579	6841	2355	1315	3670
**HIV status**						
**Negative**	6804 (88.7)	5066 (91.0)	11870 (89.7)	5233 (88.4)	2206 (89.6)	7439 (88.7)
**Positive**	864 (11.3)	504 (9.0)	1368 (10.3)	686 (11.6)	257 (10.4)	943 (11.3)

For peer review only

**Missing**		2111	2624	4735	1511	962	2473
**Number of chronic diseases**							
	**0**	4689 (77.4)	3219 (78.6)	7908 (77.9)	3633 (77)	1393 (73.5)	5026 (76.0)
	**1**	1254 (20.7)	793 (19.4)	2047 (20.2)	1001 (21.2)	455 (24.0)	1456 (22.0)
	**2**	107 (1.8)	84 (2.0)	191 (1.9)	82 (1.7)	45 (2.4)	127 (1.9)
	**3**	5 (0.1)	2 (0.0)	7 (0.1)	4 (0.1)	2 (0.1)	6 (0.1)
**Missing**		3724	4096	7820	2710	1530	4240

1. Study participants defined as those with data on disability status

Table S2. Age-specific all-cause mortality rates of men with and without disability data[1]

	With disability data			Without disability data		
**Age group**	**Number of deaths**	**Person-years**	**Rate per 1000 PY**	**Number of deaths**	**Person-years**	**Rate per 1000 PY**
**18-49**	56	10198	5.49 (4.23-7.14)	29	5502	5.27 (3.66-7.86)
**50-69**	56	2207	25.37 (19.52-32.97)	18	3623	36.23 (22.82-57.50)
**70+**	61	972	62.74 (48.81-80.63)	12	65	184.23 (104.63-324.40)

1. p value for heterogeneity between men with and without disability data = 0.94

Table S3. Poisson regression analysis of effect of graded levels of self-reported disability on all-cause mortality

	Number of deaths	Person-years	Model 1: adjusted for age only[1]	Model 2: adjusted for age, sex, & occupation[2]	Model 3: adjusted for age, sex, occupation, BMI, hypertension, diabetes, & HIV status[3,4]

For peer review only - http://bmjopen.bmj.com/site/about/guidelines.xhtml

			RR (95% CI)	p- value[5]	RR (95% CI)	p- value[5]	RR (95% CI)	p- value[5]
**Any disability**								
**No difficulty**	48	13248	1	0.12	1	0.10	1	0.09
**Some difficulty**	82	7537	1.31 (0.87-1.99)		1.51 (0.99-2.29)		1.50 (0.99-2.28)	
**A lot of difficulty/ can't do at all**	50	1937	1.71 (1.02-2.86)		1.67 (0.98-2.85)		1.71 (1.01-2.90)	

1. Adjusted for age (continuous variable); excluding participants who were missing data on occupation, BMI, hypertension, diabetes, or HIV to allow comparison with Models 2 and 3
2. Adjusted for age (continuous variable), sex (male/female), and occupation (not working, manual work, farming/fishing, non-manual work); excluding participants who were missing data on BMI, hypertension, diabetes, or HIV to allow comparison with Models 1 and 3
3. Adjusted for age (continuous variable), sex (male/female), occupation (not working, manual work, farming/fishing, non-manual work), BMI (<18.5kg/m^2, 18.5-24.9kg/m^2, 25-29.9kg/m^2, 30+kg/m^2), hypertension (defined as a measured BP >140/90 or on antihypertensive medication), diabetes (defined as fasting blood sugar >7.0 or previous diagnosis of diabetes), and HIV status (defined as a positive HIV test or diagnosis ever)
4. Likelihood ratio test for heterogeneity in the association of disability and all-cause mortality by these factors: BMI p=0.08; hypertension p=0.67; diabetes p=0.41; HIV p=0.88
5. P value is a likelihood ratio test for difference

Table S4. Poisson regression analysis of effect of graded levels of self-reported disability on all-cause mortality by hypertension status (including unknown)[1,2]

	**No hypertension**			**Hypertension**			**Unknown hypertension status**		
	Deaths	Person-years	RR (95% CI)	Deaths	Person-years	RR (95% CI)	Deaths	Person-years	RR (95% CI)
**No disability**	48	14009	1	9	1286	1	27	7099	1
**Some difficulty**	59	6719	1.48 (0.99-2.20)	34	1790	1.72 (0.82-3.61)	30	2217	1.98 (1.14-3.41)
**A lot of difficulty / can't do at all**	30	1461	1.93 (1.16-3.20)	40	844	2.27 (1.07-4.81)	51	588	5.10 (2.94-8.83)

1. All models are controlled for age (continuous variable), sex, occupation (not working, manual work, farming/fishing, non-manual work)
2. p-value for heterogeneity = 0.03

For peer review only

STROBE Statement—checklist of items that should be included in reports of observational studies

	Item No	Recommendation	
**Title and abstract**	1	(*a*) Indicate the study's design with a commonly used term in the title or the abstract	Page 1
		(*b*) Provide in the abstract an informative and balanced summary of what was done and what was found	Page 2
**Introduction**			
Background/ rationale	2	Explain the scientific background and rationale for the investigation being reported	Page 4
Objectives	3	State specific objectives, including any prespecified hypotheses	Page 5
**Methods**			
Study design	4	Present key elements of study design early in the paper	Pages 5-6
Setting	5	Describe the setting, locations, and relevant dates, including periods of recruitment, exposure, follow-up, and data collection	Pages 5-6
Participants	6	(*a*) *Cohort study*—Give the eligibility criteria, and the sources and methods of selection of participants. Describe methods of follow-up<br>*Case-control study*—Give the eligibility criteria, and the sources and methods of case ascertainment and control selection. Give the rationale for the choice of cases and controls<br>*Cross-sectional study*—Give the eligibility criteria, and the sources and methods of selection of participants	Page 6
		(*b*) *Cohort study*—For matched studies, give matching criteria and number of exposed and unexposed<br>*Case-control study*—For matched studies, give matching criteria and the number of controls per case	
Variables	7	Clearly define all outcomes, exposures, predictors, potential confounders, and effect modifiers. Give diagnostic criteria, if applicable	Pages 5-6
Data sources/ measurement	8*	For each variable of interest, give sources of data and details of methods of assessment (measurement). Describe comparability of assessment methods if there is more than one group	Pages 5-6
Bias	9	Describe any efforts to address potential sources of bias	Pages 5-6
Study size	10	Explain how the study size was arrived at	n/a
Quantitative variables	11	Explain how quantitative variables were handled in the analyses. If applicable, describe which groupings were chosen and why	Page 6
Statistical methods	12	(*a*) Describe all statistical methods, including those used to control for confounding	Page 6
		(*b*) Describe any methods used to examine subgroups and interactions	Page 6
		(*c*) Explain how missing data were addressed	Page 6
		(*d*) *Cohort study*—If applicable, explain how loss to follow-up was addressed<br>*Case-control study*—If applicable, explain how matching of cases and controls was addressed<br>*Cross-sectional study*—If applicable, describe analytical methods taking account of sampling strategy	Page 6
		(*e*) Describe any sensitivity analyses	Page 6

For peer review only

Continued on next page

For peer review only

For peer review only - http://bmjopen.bmj.com/site/about/guidelines.xhtml

**Results**			
Participants	13*	(a) Report numbers of individuals at each stage of study—eg numbers potentially eligible, examined for eligibility, confirmed eligible, included in the study, completing follow-up, and analysed	Figure 1
		(b) Give reasons for non-participation at each stage	Figure 1
		(c) Consider use of a flow diagram	Figure 1
Descriptive data	14*	(a) Give characteristics of study participants (eg demographic, clinical, social) and information on exposures and potential confounders	Table 1
		(b) Indicate number of participants with missing data for each variable of interest	Table 1
		(c) *Cohort study*—Summarise follow-up time (eg, average and total amount)	Page 8
Outcome data	15*	*Cohort study*—Report numbers of outcome events or summary measures over time	Page 8
		Case-control study—Report numbers in each exposure category, or summary measures of exposure	
		Cross-sectional study—Report numbers of outcome events or summary measures	
Main results	16	(*a*) Give unadjusted estimates and, if applicable, confounder-adjusted estimates and their precision (eg, 95% confidence interval). Make clear which confounders were adjusted for and why they were included	Table 2
		(*b*) Report category boundaries when continuous variables were categorized	
		(*c*) If relevant, consider translating estimates of relative risk into absolute risk for a meaningful time period	
Other analyses	17	Report other analyses done—eg analyses of subgroups and interactions, and sensitivity analyses	Supplementary tables
**Discussion**			
Key results	18	Summarise key results with reference to study objectives	Page 10
Limitations	19	Discuss limitations of the study, taking into account sources of potential bias or imprecision. Discuss both direction and magnitude of any potential bias	Page 11-12
Interpretation	20	Give a cautious overall interpretation of results considering objectives, limitations, multiplicity of analyses, results from similar studies, and other relevant evidence	Page 12
Generalisability	21	Discuss the generalisability (external validity) of the study results	Page 12
**Other information**			
Funding	22	Give the source of funding and the role of the funders for the present study and, if applicable, for the original study on which the present article is based	Page 14

*Give information separately for cases and controls in case-control studies and, if applicable, for exposed and unexposed groups in cohort and cross-sectional studies.

Note: An Explanation and Elaboration article discusses each checklist item and gives methodological background and published examples of transparent reporting. The STROBE checklist is best used in conjunction with this article (freely available on the Web sites of PLoS Medicine at http://www.plosmedicine.org/, Annals of Internal Medicine at http://www.annals.org/, and Epidemiology at http://www.epidem.com/). Information on the STROBE Initiative is available at www.strobe-statement.org.

For peer review only

Self-reported disability in relation to mortality in rural Malawi: a longitudinal study of over 16000 adults

Journal:	*BMJ Open*
Manuscript ID	bmjopen-2019-034802.R2
Article Type:	Original research
Date Submitted by the Author:	01-Apr-2020
Complete List of Authors:	Prynn, Josephine; University College London,<br>Dube, Albert; Malawi Epidemiology and Intervention Research Unit<br>Mkandawire, Joseph; Malawi Epidemiology and Intervention Research Unit<br>Koole, Olivier ; London School of Hygiene and Tropical Medicine Faculty of Infectious and Tropical Diseases<br>Geis, Steffen; Malawi Epidemiology and Intervention Research Unit; London School of Hygiene and Tropical Medicine Faculty of Infectious and Tropical Diseases<br>Mwaiyeghele, Elenaus; Malawi Epidemiology and Intervention Research Unit<br>Mwiba, Oddie; Malawi Epidemiology and Intervention Research Unit<br>Price, Alison; London School of Hygiene and Tropical Medicine Faculty of Infectious and Tropical Diseases<br>Kachiwanda, Lackson; Malawi Epidemiology and Intervention Research Unit<br>Nyirenda, Moffat; London School of Hygiene and Tropical Medicine Faculty of Infectious and Tropical Diseases; MRC/UVRI Uganda Research Unit On AIDS<br>Kuper, Hannah; London School of Hygiene and Tropical Medicine, Faculty of Epidemiology and Population Health<br>Crampin, Amelia; London School of Hygiene and Tropical Medicine Faculty of Infectious and Tropical Diseases; Malawi Epidemiology and Intervention Research Unit
**Primary Subject Heading**:	Epidemiology
Secondary Subject Heading:	Global health, Public health
Keywords:	EPIDEMIOLOGY, Hypertension < CARDIOLOGY, General diabetes < DIABETES & ENDOCRINOLOGY, HIV & AIDS < INFECTIOUS DISEASES, PUBLIC HEALTH

SCHOLARONE™ Manuscripts

I, the Submitting Author has the right to grant and does grant on behalf of all authors of the Work (as defined in the below author licence), an exclusive licence and/or a non-exclusive licence for contributions from authors who are: i) UK Crown employees; ii) where BMJ has agreed a CC-BY licence shall apply, and/or iii) in accordance with the terms applicable for US Federal Government officers or employees acting as part of their official duties; on a worldwide, perpetual, irrevocable, royalty-free basis to BMJ Publishing Group Ltd ("BMJ") its licensees and where the relevant Journal is co-owned by BMJ to the co-owners of the Journal, to publish the Work in this journal and any other BMJ products and to exploit all rights, as set out in our licence.

The Submitting Author accepts and understands that any supply made under these terms is made by BMJ to the Submitting Author unless you are acting as an employee on behalf of your employer or a postgraduate student of an affiliated institution which is paying any applicable article publishing charge ("APC") for Open Access articles. Where the Submitting Author wishes to make the Work available on an Open Access basis (and intends to pay the relevant APC), the terms of reuse of such Open Access shall be governed by a Creative Commons licence – details of these licences and which Creative Commons licence will apply to this Work are set out in our licence referred to above.

Other than as permitted in any relevant BMJ Author's Self Archiving Policies, I confirm this Work has not been accepted for publication elsewhere, is not being considered for publication elsewhere and does not duplicate material already published. I confirm all authors consent to publication of this Work and authorise the granting of this licence.

For peer review only

Self-reported disability in relation to mortality in rural Malawi: a longitudinal study of over 16000 adults

Corresponding Author

Dr Josephine E Prynn, University College London, London UK; Malawi Epidemiology and Intervention Research Unit (MEIRU), Lilongwe, Malawi
Josephineprynn@gmail.com
+447746427488
187 Jamaica St, London E1 0PE

Co-authors

Mr Albert Dube, Malawi Epidemiology and Intervention Research Unit (MEIRU), Lilongwe, Malawi

Dr Joseph Mkandawire, Malawi Epidemiology and Intervention Research Unit (MEIRU), Lilongwe, Malawi

Dr Olivier Koole, London School of Hygiene and Tropical Medicine, London, UK

Dr Steffen Geis, Malawi Epidemiology and Intervention Research Unit (MEIRU), Lilongwe, Malawi; London School of Hygiene and Tropical Medicine, London, UK; Institute for Medical Microbiology and Hygiene, Philipps University of Marburg, Marburg, Germany

Elenaus Mwaiyeghele, Malawi Epidemiology and Intervention Research Unit (MEIRU), Lilongwe, Malawi

Mr Oddie Mwiba, Malawi Epidemiology and Intervention Research Unit (MEIRU), Lilongwe, Malawi

Dr Alison Price, London School of Hygiene and Tropical Medicine, London, UK

Mr Lackson Kachiwanda, Malawi Epidemiology and Intervention Research Unit (MEIRU), Lilongwe, Malawi

Prof Moffat Nyirenda, Malawi Epidemiology and Intervention Research Unit (MEIRU), Lilongwe, Malawi; London School of Hygiene and Tropical Medicine, London, UK; London School of Hygiene and Tropical Medicine, London, UK

Prof Hannah Kuper, London School of Hygiene and Tropical Medicine, London, UK

Prof Amelia C Crampin, Malawi Epidemiology and Intervention Research Unit (MEIRU), Lilongwe, Malawi**;** London School of Hygiene and Tropical Medicine, London, UK

Keywords or phrases
Disability; Epidemiology of chronic non communicable diseases; epidemiology; developing countries; mortality

Word count: 3121

For peer review only

Abstract

Objectives We investigated whether self-reported disability was associated with mortality in adults in rural Malawi.

Setting Karonga Health and Demographic Surveillance Site (HDSS), Northern Malawi

Participants All adults aged 18 and over residing in the HDSS were eligible to participate. During annual censuses in 2014 and 2015, participants were asked if they experienced difficulty in any of six functional domains and were classified as having disabilities if they reported "a lot of difficulty" or "can't do at all" in any domain. Mortality data was collected until December 31st, 2017. 16748 participants (10153 women and 6595 men) were followed up for a median of 29 months.

Primary and secondary outcome measures We used Poisson regression to examine the relationship between disability and all-cause mortality adjusting for confounders. We assessed whether this relationship altered in the context of obesity, hypertension, diabetes, or HIV. We also evaluated whether mortality from NCDs was higher among people who had reported disability, as determined by verbal autopsy.

Results: At baseline, 7.6% reported a disability and the overall adult mortality rate was 9.4/1000 person-years. Adults reporting disability had an all-cause mortality rate 2.70 times higher than those without, and mortality rate from non-communicable disease 2.33 time higher than those without.

Conclusions Self-reported disability predicts mortality at all adult ages in rural Malawi. Interventions to improve access to healthcare and other services are needed.

For peer review only

For peer review only - http://bmjopen.bmj.com/site/about/guidelines.xhtml

Strengths and Limitations of This Study

- We were able to use mortality data collected through a well-functioning health and demographic surveillance survey in a country without complete vital registration
- The large sample size allowed us to examine the relationship between disability and mortality over a relatively short follow-up period
- This is the first study to examine whether disability as defined by the Washington Group questions is predictive of mortality, so contributes substantially to the existing literature on this topic
- A limitation of the study is that we have considerable missing data in chronic health states (BMI, hypertension, diabetes, HIV), which limits our understanding of how these are related to disability and mortality, and that verbal autopsies were used to establish cause of death

Abbreviations

Body mass index (BMI)

Health and Demographic Surveillance Site (HDSS)

Non-communicable diseases (NCDs)

Sub-Saharan Africa (SSA)

Sustainable Development Goals (SDGs)

For peer review only - http://bmjopen.bmj.com/site/about/guidelines.xhtml

Introduction

Disability is a complex concept encompassing long-term physical, mental, intellectual or sensory impairments which, in interaction with personal and environmental factors, may limit people's participation in society on an equal basis with others.[1] The World Health Organisation (WHO) estimates that one billion people globally have a disability,[1] and the number is expected to rise further as the global population continues growing, life expectancy rises, and average age increases. People with disabilities are being "left behind" in terms of inclusion in education, employment, and social life,[1] and are at high risk of poverty.[2] Consequently, disability is recognised as an important development issue, and is explicitly referenced within five of the Sustainable Development Goals (SDGs).[3]

Disability is expected to be linked to increased mortality through various pathways. Both disability and mortality are related to ageing and poverty, and so associations may arise through confounding.[1, 2] An underlying disease may also cause both disability and mortality, for instance diabetes can lead to impairments (e.g. visual impairment from diabetic retinopathy) and premature death. People with disabilities may find it more difficult to seek health-care due to a range of barriers,[1] thereby increasing their mortality risk. They may also have unhealthier behaviours, on average, regarding smoking, physical activity, and diet, increasing their vulnerability to obesity, non-communicable diseases (NCDs) and mortality.[4] There is also evidence that people with disabilities are more vulnerable to HIV and other infectious diseases, due to their marginalised position in society.[5] Certain impairments, such as mobility or cognitive impairments, can increase frailty, depression and functional difficulties, all linked to increased mortality.[6-8] Data are, however, lacking on the link between disability and mortality, particularly from low- and middle-income settings, where over 80% of people with disabilities reside.[9] One reason for the lack of data has been due to lack of assessment of disability in surveys and cohorts. The debate has raged as to how disability should be defined and measured, yet scales are needed to estimate the prevalence and impacts of disability, and to allow international data comparisons. Consensus is growing on the use of the Washington Group (WG) Short Set to collect Disability Statistics,[10] which will improve data comparability. The WG questions focus on difficulties in functioning in 6 different domains related to activities (e.g. walking) and participation (e.g. performing usual activities).

For peer review only - http://bmjopen.bmj.com/site/about/guidelines.xhtml

For peer review only

The objective of this study was to investigate the relationship between self-reported disability and mortality among adults in rural Malawi. A secondary objective was to assess whether the effect of disability on mortality varies with co-existence of obesity or a chronic disease (hypertension, diabetes, or HIV), and by cause of death.

Methods

Setting and data collection

The Karonga Health and Demographic Surveillance Site (HDSS) in Northern Malawi comprises a population of around 40,000 individuals, which is largely representative of the rural Malawian population in terms of age and sex structure.[11, 12] Census information is collected on the population annually, along with continuous reporting of births, deaths, and migration by community key informants. Verbal autopsy is done after every death using a semi-structured interview of a family member using an adaptation of a WHO instrument.[13] Two clinically trained reviewers independently assign cause of death based on this interview. In case of disagreement, a third reviewer arbitrates.

Since 2014, the WG short set questions on disability have been added to the annual census questionnaire, alongside the existing questions on demographic, health, and social indicators. The disability questions were only asked if the participant was physically seen by the fieldworker, although they could be answered through a proxy, after obtaining written consent or assent. Therefore, no disability data was collected from anyone away from home on the day of the census.

The questions, translated into the local language of Chitumbuka, are:

- Do you have difficulty seeing, even if wearing glasses?
- Do you have difficulty hearing, even if using a hearing aid?
- Do you have difficulty walking or climbing steps?
- Do you have difficulty remembering or concentrating?
- Do you have difficulty (with self-care such as) washing all over or dressing?

For peer review only

- Using your usual (customary) language, do you have difficulty communicating, for example understanding or being understood?

In this analysis, we used data on adults aged 18 and over from the first two consecutive census rounds to include the disability questions. The first was in 2014-2015 (Round 1), the second in 2015-2016 (Round 2). Baseline disability status was taken from the Round 1 census data where possible. For participants with no disability data from Round 1, data from Round 2 was used. Anyone who moved into the HDSS between Rounds 1 and 2, or turned 18 between Rounds 1 and 2, and answered disability questions at Round 2 was also included. Other socio-demographic information was taken from the same interview as the disability data. Follow-up was undertaken until 31st December 2017.

Data on body mass index (BMI), hypertension, and diabetes were gathered from a survey of all adults performed within the HDSS population in 2013-2015 on prevalence of major NCDs and their risk factors.[14] In this screening, height and weight were measured twice, and the mean was used to calculate BMI. Participants were asked if they had previously been diagnosed with hypertension or diabetes, and whether they were taking any regular medication. Resting blood pressure was measured three times with 5 minutes in between, and a mean of the second and third measures were used. Blood was taken for plasma glucose measurement after at least 8 hours fasting. This data was collected a mean of 1.2 years prior to the disability data (maximum 3.5 years).

Data on HIV status was collated from numerous sources including an HIV serosurvey in 2011 and the 2013-2015 NCD survey. Data on new HIV diagnoses was also collected from consenting participants at government clinics within the HDSS. Participants were categorised as HIV-positive if they had ever reported a diagnosis of HIV or had a positive antibody test. They were categorised as negative for 4 years after a negative HIV-test, after which time their status was categorised as missing in case of a new infection in the interim.

Variables

Age was grouped into categories of 18-34, 35-44, 45-54, 55-64, 65-69, 70-74, 75-79, and 80+ years; narrower age bands were chosen at higher ages as self-reported disability is strongly associated with older age. Education level was defined as no education, primary (including partially-completed), secondary (including partially-completed), and tertiary. Occupation was categorised into not working (including unemployed, unable to work, and retired), manual

For peer review only - http://bmjopen.bmj.com/site/about/guidelines.xhtml

work, farming or fishing, and non-manual work (including professional work). Participants were defined as in a union if they were married or co-habiting, and not if single, divorced, or widowed.

Categories of BMI were <18.5kg/m² (underweight), 18.5-24.9kg/m² (healthy weight), 25-29.9kg/m² (overweight), and 30+kg/m² (obese). Hypertension was defined as systolic blood pressure ≥140mmHg, diastolic blood pressure of ≥90mmHg, or use of antihypertensive medications. Diabetes was defined as fasting blood sugar ≥7.0mmol/L or a self-reported diagnosis of diabetes. Disability was defined as answering "a lot of difficulty" or "can't do at all" in any domain.

Cause of death among adults was broadly categorised into communicable disease, NCDs, maternal death, external (including injury and poisoning), and unspecifiable/other.

Statistical analysis

Prevalence of self-reported disability was calculated with 95% confidence intervals by socio-demographic and health characteristics. Poisson regression analysis was used to calculate adult mortality rate ratios, comparing people reporting "some difficulty" and "a lot of difficulty or can't do at all" with "no difficulty" in each disability domain. For these analyses, individuals contributed exposure time during their residence in the HDSS from the date the disability survey was completed, until the earliest of 31st December 2017, death, or out-migration. Returning and repeat migrants only contributed person-years while resident in the HDSS.

Age and sex were included *a priori* in the adjusted model, and baseline occupation, education level, and union status were sequentially added to the model to check for confounding. Any variable that altered the rate ratio more than 10% was kept in the adjusted model. Each of obesity, hypertension, diabetes and HIV status at baseline were also added to the models to check for confounding or effect modification. Cause-specific mortality was also calculated. We performed complete case analysis, so that any participants with missing data for any of the variables in the model were excluded. Sensitivity analyses, including an "unknown" category for BMI, hypertension, diabetes, and HIV were also performed.

All significance tests were likelihood-ratio tests.

Ethical approval

For peer review only - http://bmjopen.bmj.com/site/about/guidelines.xhtml

Ethical approval for the HDSS census rounds and NCD survey was granted by the National Health Sciences Research Committee (NHSRC) (protocol numbers #419 and #1072 respectively), and by the London School of Hygiene and Tropical Medicine (LSHTM) (protocol numbers #5081 and #6303 respectively).

Patient and public involvement

MEIRU works closely with the community in which this research was conducted. Regular meetings with senior community-members take place to ensure that study objectives align with the priorities of the community, and that the methodology and procedures are appropriate and acceptable. Research findings are disseminated similarly.

For peer review only

Results

Of 17973 adult (≥18 years) residents seen at Round 1, 10855 (60.4%) responded to questions on disability. A further 5893 adults not included at Round 1 (because they were under 18 years, not living in the area at Round 1, or missed) provided disability data at Round 2, giving 16748 participants with baseline disability data (**Figure 1**).

Those without disability data were more likely to be younger (22.3% of 18-39-year-olds compared to 10.8% of those aged 40+) and male (29.4% of men compared to 9.5% of women). Baseline characteristics of the census population compared to those included in the study are in **Table S1**. Participants with disability data had a median follow-up of 29 months (interquartile range 20-33 months). Migration out of the HDSS led to censoring of data in 1574 (9.5%) of participants. Individuals who migrated were more likely to be young and have higher educational attainment.

Table 1 shows that most participants were under age 35, and 76.1% of women and 62.5% of men were farmers or fishermen. The prevalence of obesity was higher in women than men (7.4% vs. 1.3%), whereas prevalence of hypertension, diabetes, and HIV were similar among men and women. Hypertension, diabetes, and HIV variables were not available for at least 30% of participants.

Prevalence of self-reported disability in one or more domain was 7.6% and strongly associated with increasing age (p test-for-trend <0.001). Adjusted for age, people with lower levels of education and those not working were more

For peer review only - http://bmjopen.bmj.com/site/about/guidelines.xhtml

likely to report disability, and prevalence was higher among participants with obesity, hypertension, or diabetes. People living with HIV reported a similar prevalence of disability to those who were HIV negative.

There were 328 deaths in 36019 person-years of follow-up. The adult mortality rate was 9.1/1000 person-years (95% CI 8.2-10.1) and higher among men (12.9/1000 person-years; 95%CI 11.1-15.0) than women (6.8/1000 person-years; 95% CI 5.8-8.0) including when controlling for age (rate ratio 1.75; 95% CI 1.44-2.14). Sensitivity analyses comparing all-cause mortality rates in men without disability data to those with disability data suggested no material difference stratified by age (**Table S2**).

Table 2 shows that compared with adults reporting “no difficulty” in any disability domain, those reporting “some difficulty”, and “a lot of difficulty or can’t do at all” had greater mortality (RR 1.39; 95% CI 1.03-1.88 and RR 2.70; 95% CI 1.91-3.82, respectively). Except for difficulty seeing and hearing, self-reported disability in any of the domains was strongly associated with increased mortality when adjusted for age, and the association remained when also adjusted for sex and occupation. For every domain, reporting disability (“a lot of difficulty” or “can’t do at all”) was associated with a higher mortality than answering “some difficulty”. Disability in increasing numbers of domains was strongly associated with mortality. The relationship between any self-reported disability and all-cause mortality did not vary by sex or age group (**Table 3**); and none of BMI, hypertension, diabetes, or HIV were confounders or effect modifiers of the relationship (**Table S3**). Sensitivity analysis including a “missing” category for hypertension did show heterogeneity by hypertension status (p=0.03): the relationship between disability and mortality was stronger among those with unknown hypertension status (**Table S4**). Including a “missing” category for BMI, diabetes, and HIV did not show any heterogeneity.

Table 4 shows that mortality from non-communicable diseases was higher among people living with disability than those without disability. No difference was seen for mortality from HIV or other communicable diseases.

Discussion

For peer review only - http://bmjopen.bmj.com/site/about/guidelines.xhtml

Our findings show that self-reported disability in adults is strongly associated with increased all-cause mortality in rural Malawi. Furthermore, a stepwise increase in risk was observed with increasing levels of difficulties in functioning and numbers of domains affected. Individuals reporting difficulty in walking and self-care were particularly at risk. The co-existence of obesity or chronic disease did not materially alter estimates of effect, and the magnitude of the association was consistent across age- and sex- groups. People dying from NCDs were more likely to have been living with disability before death than those dying from HIV.

Our findings are similar to those observed elsewhere in sub-Saharan Africa (SSA). A study in Tanzanian adults aged 70+ also found a stepwise increase in mortality with increasing disability severity.[15] In South African adults aged 50+, severe functional disability was associated with a mortality rate ratio of 1.92 in women and 2.80 in men, compared to those with least functional disability.[16] In Malawi, Payne et al described an increasing mortality risk with increasingly severe disability among adults aged 45 and over, compared with those without disability.[17] None of these studies used the WG questions, however.

The association between disability and mortality has also been observed in high-income countries (HIC).[18-21] This is seen even after adjusting for a wide range of comorbidities,[22-24] suggesting that the association between disability and mortality is not solely due to conditions predisposing to both disability and death. Nonetheless, contexts differ widely between high-resource settings and rural Malawi hence these findings may not be generalizable to our setting. In HIC, improved access to healthcare means that many more people live with treated chronic disease over many years, as opposed to in a low -income country (LIC) like Malawi, where people may be more likely to die early from disabling disease.[14, 25] Even access to safe water and sanitation services can be profoundly affected by disability in LIC including Malawi,[26, 27] which is an important risk factor for disease.

We observed a higher prevalence of disability in women than men at all ages, yet mortality rates were consistently higher in men than women across all adult age groups, irrespective of disability. This apparent “male-female health survival paradox” has been seen in a wide variety of settings, using a range of disability definitions.[15, 16, 23]

The population in Malawi is ageing, as in much of SSA.[28] As such, the very strong positive correlation between increasing age and prevalence of self-reported disability portends a growing prevalence of disability in the

For peer review only - http://bmjopen.bmj.com/site/about/guidelines.xhtml

population in coming decades. The extent to which increasing life expectancy leads to an increased number of years lived with disability, or whether onset of disability will be delayed with a healthier life-course leading to increased life expectancy, is not yet understood.[29]

A major strength of our study is the available mortality data from a long-established HDSS, when vital registration is very limited in Malawi.[30] Moreover, the large sample size and low loss to follow-up allows meaningful mortality analyses despite a relatively short follow-up time. Disability is not a static state: people move considerably between disability states over time.[17, 31] Despite this, a one-off self-report of disability put individuals at a much-increased risk of death in the following 2 years. As further disability and mortality data is collected with time, we will be able to examine mortality over a longer follow-up time and consider self-reported disability as a time-varying exposure in future analyses. Our study adds considerably to the very limited literature examining mortality with disability in this context and is the first to examine whether disability as defined by the WG questions is predictive of mortality.

Our study has several limitations. The WG questions do not capture a comprehensive picture of disability, for example common impairments such as affective disorders and chronic pain are not included. Furthermore, the use of self-report relies on participants having comparable interpretations of what constitutes “some difficulty” versus “a lot of difficulty”. Self-report also requires people to be able to understand the questions and communicate their response, so there is a risk that some participants would have been unable to self-identify. However, by allowing proxy responses where necessary, this will have been partially mitigated. We have limited information on underlying health conditions in the population and our data on hypertension, diabetes, BMI, and HIV were not measured concurrently with disability. If participants were to change category in the intervening time misclassification could occur, but as these are all, in the main, long-term health states, it is unlikely that this will have affected a substantial number of participants. We had considerable missing data on hypertension, diabetes, and HIV status, and our data may have been more likely to capture positive cases than negative. Moreover, it is likely that data were not missing at random, increasing the chance of bias in results. For hypertension and diabetes, the data was missing mainly on younger, working men, who are likely to have lower prevalence than the general population.[30] For HIV status some data is from new diagnoses made at health centres, where people may present if they feel they are at risk of being HIV positive. Participants with missing data on hypertension demonstrated a stronger relationship between disability and mortality than those with known hypertension or known normotension,

For peer review only - http://bmjopen.bmj.com/site/about/guidelines.xhtml

suggesting that the hypertension data is not missing at random. These potential biases limit our understanding of the relationship of these individual conditions with disability and mortality, as well as the composite measure of "number of chronic conditions", and more research is needed to see whether our findings are replicated in both this and other settings. Furthermore, cause of death was collected through verbal autopsy, which is subject to bias. Our pragmatic approach to only collect disability reports on individuals found at home during our annual census means that older men were over-represented in our sample compared to the census. Consequently, the increased mortality in men compared to women seen in all age groups could be suspected to be due to a "healthy worker" effect as more healthy men who will likely have lower mortality risk work far from the household in paid employment, while this pattern is not seen in women. However, we found that the relationship of disability and mortality did not vary by age group and that there were no differences in age-specific mortality by disability status when stratified by age. Particularly among younger men, the mortality rate was very similar among those included in this analysis and those excluded.

Conclusions

Disability is commonly reported and likely to increase in Malawi in coming decades as the population ages and NCDs become increasingly prevalent. Self-reported disability is associated with significantly increased mortality, which seems to be driven particularly by an increased mortality rate from non-communicable diseases. While more research is urgently needed to help understand the mechanism behind this relationship, this needs to be concurrent with active interventions to improve access to healthcare and other services, particularly for people with disabilities, if the commitment to "leave no-one behind"[32] in the SDGs is to be realised.

References

1. World Health Organisation, *World Report on Disability*. 2011.
2. Banks, L.M., H. Kuper, and S. Polack, *Poverty and disability in low- and middle-income countries: A systematic review.* PLOS ONE, 2017. **12**(12): p. e0189996.

For peer review only - http://bmjopen.bmj.com/site/about/guidelines.xhtml

3. United Nations. *Sustainable Development Goals (SDGs) and Disability*. 16th April 2019]; Available from: https://www.un.org/development/desa/disabilities/about-us/sustainable-development-goals-sdgs-and-disability.html.
4. Hashemi, G., M. Wickenden, and H. Kuper, *SDGs, Inclusive Health and the path to Universal Health Coverage.* Disability and the Global South, 2017. **4**(1): p. 23.
5. De Beaudrap, P., M. Mac-Seing, and E. Pasquier, *Disability and HIV: a systematic review and a meta-analysis of the risk of HIV infection among adults with disabilities in Sub-Saharan Africa.* AIDS Care, 2014: p. 1-10.
6. Liljas, A.E.M., et al., *Self-reported vision impairment and incident prefrailty and frailty in English community-dwelling older adults: findings from a 4-year follow-up study.* J Epidemiol Community Health, 2017. **71**(11): p. 1053-1058.
7. Ribeiro, M.V., et al., *Association between visual impairment and depression in the elderly: a systematic review.* Arq Bras Oftalmol, 2015. **78**(3): p. 197-201.
8. Cimarolli, V.R. and D.S. Jopp, *Sensory impairments and their associations with functional disability in a sample of the oldest-old.* Qual Life Res, 2014. **23**(7): p. 1977-84.
9. World Health Organisation, *Concept Note: World Report on Disability and Rehabilitation*. 2007.
10. *Washington Group on Disability Statistics* Available from: http://www.washingtongroup-disability.com/.
11. Malawi National Statistical Office, *Population and Housing Census Results* 2008.
12. Dube, A., et al., *Profile: The Karonga Health and Demographic Surveillance System.* International Journal of Epidemiology, 2012. **41**(3): p. 676-685.
13. World Health Organisation. *Verbal autopsy standards: ascertaining and attributing causes of death*. 2018 14th July 2019]; Available from: https://www.who.int/healthinfo/statistics/verbalautopsystandards/en/.
14. Price, A.J., et al., *Prevalence of obesity, hypertension, and diabetes, and cascade of care in sub-Saharan Africa: a cross-sectional, population-based study in rural and urban Malawi.* The Lancet Diabetes & Endocrinology, 2018. **6**(3): p. 208-222.
15. Gray, W.K., et al., *Rates and predictors of three-year mortality in older people in rural Tanzania.* Archives of Gerontology and Geriatrics, 2016. **62**(Supplement C): p. 36-42.
16. Gómez-Olivé, F.X., et al., *Social conditions and disability related to the mortality of older people in rural South Africa.* International Journal of Epidemiology, 2014. **43**(5): p. 1531-1541.
17. Payne, C., J. Mkandawire, and H.-P. Kohler, *Disability Transitions and Health Expectancies among Adults 45 Years and Older in Malawi: A Cohort-Based Model.* PLoS Med, 2013. **10**(5): p. e100435.
18. Park, J.M., et al., *Disparities in mortality by disability: an 11-year follow-up study of 1 million individuals.* International Journal of Public Health, 2017. **62**(9): p. 989-996.
19. Björkenstam, C., et al., *Diagnosis-specific disability pension and risk of all-cause and cause-specific mortality – a cohort study of 4.9 million inhabitants in Sweden.* BMC Public Health, 2014. **14**(1): p. 1247.
20. Karlsson, N.E., et al., *Mortality in relation to disability pension: Findings from a 12-year prospective population-based cohort study in Sweden.* Scandinavian Journal of Public Health, 2007. **35**: p. 341-347.
21. Forman-Hoffman, V.L., et al., *Disability status, mortality, and leading causes of death in the United States community population.* Med Care, 2015. **53**(4): p. 346-54.
22. Hennessy, S., et al., *Disability Stage is an Independent Risk Factor for Mortality in Medicare Beneficiaries Aged 65 Years and Older.* PM&R, 2015. **7**(12): p. 1215-1225.
23. Pongiglione, B., et al., *Disability and all-cause mortality in the older population: evidence from the English Longitudinal Study of Ageing.* European Journal of Epidemiology, 2016. **31**(8): p. 735-746.
24. Majer, I.M., et al., *Mortality risk associated with disability: a population-based record linkage study.* Am J Public Health, 2011. **101**(12): p. e9-15.
25. Dewhurst, F., et al., *The prevalence of disability in older people in Hai, Tanzania.* Age Ageing, 2012. **41**(4): p. 517-23.
26. Groce, N., et al., *Water and sanitation issues for persons with disabilities in low- and middle-income countries: a literature review and discussion of implications for global health and international development.* Journal of Water and Health, 2011. **9**(4): p. 617.
27. White, S., et al., *A qualitative study of barriers to accessing water, sanitation and hygiene for disabled people in Malawi.* PLOS ONE, 2016. **11**(5).
28. Division, U.N.D.P., *World Population Prospects: The 2017 Revision*. 2017.
29. Beard, J.R., et al., *The World report on ageing and health: a policy framework for healthy ageing.* Lancet, 2016. **387**: p. 2145-54.

For peer review only - http://bmjopen.bmj.com/site/about/guidelines.xhtml

30. Crampin, A.C., et al., *Hypertension and diabetes in Africa: design and implementation of a large population-based study of burden and risk factors in rural and urban Malawi.* Emerg Themes Epidemiol, 2016. **13**: p. 3.
31. Prynn, J.E., et al., *Self-reported disability in rural Malawi: prevalence, incidence, and relationship to chronic disease [version 1; peer review: awaiting peer review].* Wellcome Open Research, 2019. **4**(90).
32. United Nations. *The Sustainable Development Goals Report*. 2016 16th August 2018]; Available from: https://unstats.un.org/sdgs/report/2016/leaving-no-one-behind.

Footnotes

Author Contributions

Conceptualisation: OK, AP, HK, ACC

Funding acquisition: MN, ACC

Design: AD, SG, AP

Project administration: AD, JM, EM, OM

Supervision of data collection: AD. OK, SG, EM, AP, LK

Data curation: OM

Analysis: JEP, ACC

Original draft preparation: JEP, HK, ACC

Review and editing of manuscript: JEP, AD, JM, OK, SG, EM, OM, AP, LK, MN, HK, ACC

Funding statement

This work was supported by the Wellcome Trust, grant number #098610

Competing interests

There were no competing interests for any author

For peer review only

Data Sharing Agreement

Data from the NCD survey is available from LSHTM Data Compass: Malawi Epidemiology and Intervention Research Unit Non-Communicable Disease Survey data, 2013–2017. https://doi.org/10.17037/DATA.0000096136. Data are available under the terms of the Creative Commons Attribution 3.0 International license (CC-BY 3.0).

Summary demographic datasets are publicly available through the INDEPTH iShare platform.

Longitudinal data (demographic surveillance episodes and linked rounds of disability questionnaires) cannot be sufficiently de-identified for public availability. Application may be made for access through the MEIRU director (mia.crampin@lshtm.ac.uk) or data scientist Chifundo Kanjala (chifundo.kanjala@lshtm.ac.uk). Those wishing to access the data will need to provide a brief proposal for what the data will be used for as a condition of access.

For peer review only

Table 1. Baseline characteristics of participants with baseline disability data and proportion with self-reported disability[1]

	Women			Men			Total		
	n	Number reporting disability (%)	p-value[2]	n	Number reporting disability (%)	p-value[2]	n	Number reporting disability (%)	p-value[2]
**Overall**	10153	857 (8.4)	-	6595	420 (6.4)	-	16748	1277 (7.6)	-
**Age group**									
18-34	5503	167 (3.0)	<0.001*	3378	73 (2.2)	<0.001*	8881	240 (2.7)	<0.001*
35-44	1914	103 (5.4)		1339	48 (3.6)		3253	151 (4.6)	
45-54	1100	122 (11.1)		786	55 (7.0)		1886	177 (9.4)	
55-64	773	116 (15.0)		486	64 (13.2)		1259	180 (14.3)	
65-69	258	54 (20.9)		175	25 (14.3)		433	79 (18.2)	
70-74	212	75 (35.4)		133	38 (28.6)		345	113 (32.8)	
75-79	189	94 (49.7)		138	45 (32.6)		327	139 (42.5)	
80+	204	126 (61.8)		160	72 (45.0)		364	198 (54.4)	
**Education level[3]**									
None	378	117 (31.0)	0.002*	98	27 (27.6)	0.06*	476	144 (30.3)	<0.001*
Some primary/completed primary	6603	632 (9.6)		3284	269 (8.2)		9887	901 (9.1)	
Some secondary/completed secondary	2633	82 (3.1)		2707	99 (3.7)		5340	181 (3.4)	
Tertiary	469	15 (3.2)		467	24 (5.1)		936	39 (4.2)	
**Occupation[4]**									
Not working	1013	202 (19.9)	<0.001	985	106 (10.8)	<0.001	1998	308 (15.4)	<0.001
Manual	143	5 (3.5)		745	28 (3.8)		888	33 (3.7)	
Farmer/fisherman	7637	567 (7.4)		4092	256 (6.3)		11729	823 (7.0)	
Non-manual/ business/ professional	1249	62 (5.0)		724	26 (3.6)		1973	88 (4.5)	
**Union status[5]**									
Not in a union	3381	479 (14.2)	<0.001	1933	97 (5.0)	0.001	5314	576 (10.8)	<0.001
In a union	6768	378 (5.6)		4659	323 (6.9)		11427	701 (6.1)	
**BMI (kg/m^2)[6]**									

For peer review only

For peer review only - http://bmjopen.bmj.com/site/about/guidelines.xhtml

<18.5	669	61 (9.1)	0.005	601	57 (9.5)	0.38	1270	118 (9.3)	<0.001
18.5-24.9	6074	423 (7.0)		4847	263 (5.4)		10921	686 (16.3)	
25-29.9	1869	176 (9.4)		497	50 (10.1)		2366	226 (9.6)	
30+	689	90 (13.1)		80	10 (12.5)		769	100 (13.0)	
**Hypertension[7]**									
No hypertension	6069	416 (6.9)	0.69	3599	200 (5.6)	0.05	9668	616 (6.4)	0.05
Hypertension	1011	242 (23.9)		633	110 (17.4)		1644	352 (21.4)	
**Diabetes[8]**									
No diabetes	6189	535 (8.6)	0.22	3582	259 (7.2)	0.007	9771	794 (8.1)	0.008
Diabetes	109	25 (22.9)		69	19 (27.5)		178	44 (24.7)	
**HIV status[9]**									
Negative	6128	490 (8.0)	0.69	3070	218 (7.1)	0.60	9198	708 (7.7)	0.90
Positive	907	70 (7.7)		443	38 (8.6)		1350	108 (8.0)	
**Number of chronic diseases[10,11]**									
0	4243	261 (6.2)	0.77*	2054	102 (5.0)	0.002*	6297	363 (5.8)	0.02*
1	1166	170 (14.6)		620	91 (14.7)		1786	261 (14.6)	
2	100	19 (19.0)		69	14 (20.3)		169	33 (19.5)	
3	5	1 (20.0)		2	1 (50.0)		7	2 (28.6)	

1. Defined as answering "a lot of difficulty" or "can't do at all" to at least one disability domain
2. p-value for difference using likelihood ratio test, values marked as * (age, education level, number of chronic diseases) p-value for trend. For all variables other than age group this is controlled for age (as a continuous variable)
3. Education level: No data on 70 women and 39 men
4. Occupation: No data on 111 women and 49 men
5. Union status: No data on 4 women and 3 men
6. BMI: No data on 872 women and 570 men
7. Hypertension: No data on 3073 women and 2363 men
8. Diabetes: No data on 3855 women and 2944 men
9. HIV status: No data on 3118 women and 3082 men
10. Chronic diseases included in this variable: hypertension, diabetes, HIV
11. Number of chronic diseases variable: No data on 4639 women and 3850 men (only participants with data on all 3 chronic diseases included)

For peer review only - http://bmjopen.bmj.com/site/about/guidelines.xhtml

Table 2. Poisson regression analysis of effect of graded levels of self-reported disability on all-cause adult mortality

	Number of deaths	Person-years	Model 1: adjusted for age only[1]		Model 2: adjusted for age, sex, & occupation[2]	
			RR (95% CI)	p- value[3]	RR (95% CI)	p-value[3]
**Difficulty seeing**						
No difficulty	165	28106	1			
Some difficulty	107	6316	1.02 (0.79-1.34)	0.38	1.08 (0.82-1.41)	0.85
A lot of difficulty/ can't do at all	49	1255	1.20 (0.84-1.73)		1.01 (0.7-1.46)	
**Difficulty hearing**						
No difficulty	296	33922	1			
Some difficulty	39	1480	1.20 (0.85-1.71)	0.05	1.11 (0.78-1.58)	0.19
A lot of difficulty/ can't do at all	13	275	1.79 (1.01-3.15)		1.51 (0.85-2.68)	
**Difficulty walking**						
No difficulty	157	30222	1			
Some difficulty	79	4175	1.39 (1.04-1.88)	<0.001	1.57 (1.15-2.13)	<0.001
A lot of difficulty/ can't do at all	85	1278	3.40 (2.48-4.66)		3.20 (2.27-4.51)	
**Difficulty remembering**						
No difficulty	204	29608	1		1	
Some difficulty	79	5384	1.06 (0.81-1.39)	<0.001	1.17 (0.89-1.54)	<0.001
A lot of difficulty/ can't do at all	37	650	2.67 (1.84-3.85)		2.55 (1.74-3.73)	
**Difficulty communicating**						
No difficulty	287	35235	1		1	
Some difficulty	23	332	3.11 (2.01-4.8)	<0.001	2.26 (1.44-3.53)	<0.001
A lot of difficulty/ can't do at all	10	82	8.81 (4.67-16.59)		5.33 (2.78-10.21)	

For peer review only

**Difficulty with self-care**						
No difficulty	234	33850	1	<0.001	1	<0.001
Some difficulty	47	1306	1.84 (1.33-2.56)		1.74 (1.24-2.45)	
A lot of difficulty/ can't do at all	38	503	3.75 (2.62-5.38)		3.14 (2.15-4.6)	
**Any disability**						
No difficulty	84	22197	1	<0.001	1	<0.001
Some difficulty	122	10645	1.39 (1.03-1.88)		1.61 (1.18-2.19)	
A lot of difficulty/ can't do at all	115	2839	2.70 (1.91-3.82)		2.65 (1.84-3.8)	
**Disability in multiple domains**						
No disability	206	32848	1	<0.001	1	<0.001
Disability in 1 domain	51	2018	1.60 (1.15-2.21)		1.51 (1.08-2.11)	
Disability in 2 domains	31	543	2.39 (1.59-3.59)		2.07 (1.35-3.17)	
Disability in 3 domains	19	120	3.71 (2.26-6.09)		3.06 (1.83-5.14)	
Disability in 4+ domains	14	79	5.64 (3.19-9.96)		4.33 (2.39-7.88)	

1. Controlled for age (continuous variable)
2. Controlled for age (continuous variable), sex, and occupation (not working, manual work, farming/fishing, non-manual work)
3. Likelihood ratio test for difference

For peer review only - http://bmjopen.bmj.com/site/about/guidelines.xhtml

Table 3. Poisson regression of disability[1] on all-cause adult mortality stratified by age group and sex[1]

Age group		Women					Men				
		No. of deaths	PY[3]	Mortality rate per 1000 PY (95% CI)	RR[4] (95%CI)	p-value	No. of deaths	PY[4]	Mortality rate per 1000 PY (95% CI)	RR[3] (95% CI)	p-value
**18-49**	No disability	40	16741	2.39 (1.75-3.26)	1	0.04	51	9870	5.17 (3.93-6.80)	1	0.10
	Disability	6	739	8.12 (3.65-18.09)	2.76 (1.16-6.58)		5	324	15.43 (6.42-37.06)	2.37 (0.94-5.97)	
**50-69**	No disability	25	3169	7.89 (5.33-11.67)	1	0.002	44	1952	22.54 (16.78-30.29)	1	0.06
	Disability	14	566	24.74 (14.65-41.77)	3.00 (1.55-5.79)		12	255	46.98 (26.68-82.73)	1.93 (1.02-3.65)	
**70+**	No disability	18	749	24.04 (15.15-38.15)	1	<0.001	29	636	45.62 (31.70-65.64)	1	0.009
	Disability	52	673	77.30 (58.90-101.45)	2.76 (1.61-4.75)		32	337	95.07 (67.23-134.44)	1.98 (1.19-3.30)	
**All**	No disability	83	20659	4.02 (3.24-4.98)	1	<0.001	124	12458	9.95 (8.35-11.87)	1	0.001
	Disability	72	1977	36.42 (28.91-45.88)	2.89 (1.20-4.19)		49	916	53.49 (40.42-70.77)	1.87 (1.30-2.71)	

1. Disability is defined as reporting "a lot of difficulty" or "can't do at all" in any of the functional domains
2. p-value for heterogeneity between age-sex groups = 0.94
3. Person-years
4. Rate ratio, adjusted for age (as a continuous variable)

For peer review only

Table 4. Cause-specific mortality rate ratio among those living with disability prior to death compared to those not living with disability

Cause of death[1]	Number who died		Mortality rate ratio[2]	p-value
	Disability	No disability		
**Communicable disease (excluding HIV)**	17	12	1.42 (0.62-3.26)	0.41
**HIV**	52	10	1.80 (0.89-3.65)	0.10
**Non-communicable disease**	80	64	2.33 (1.60-3.38)	<0.01
**Maternal**	1	0	-	-
**External**	19	2	1.22 (0.32-4.65)	0.77
**Unspecified/other**	7	10	3.92 (1.30-11.81)	0.02

1. **Cause of death** attributed by verbal autopsy
2. **Adjusted for age** (as a continuous variable)

Figures

Figure 1. Flow chart of participation

For peer review only

For peer review only - http://bmjopen.bmj.com/site/about/guidelines.xhtml

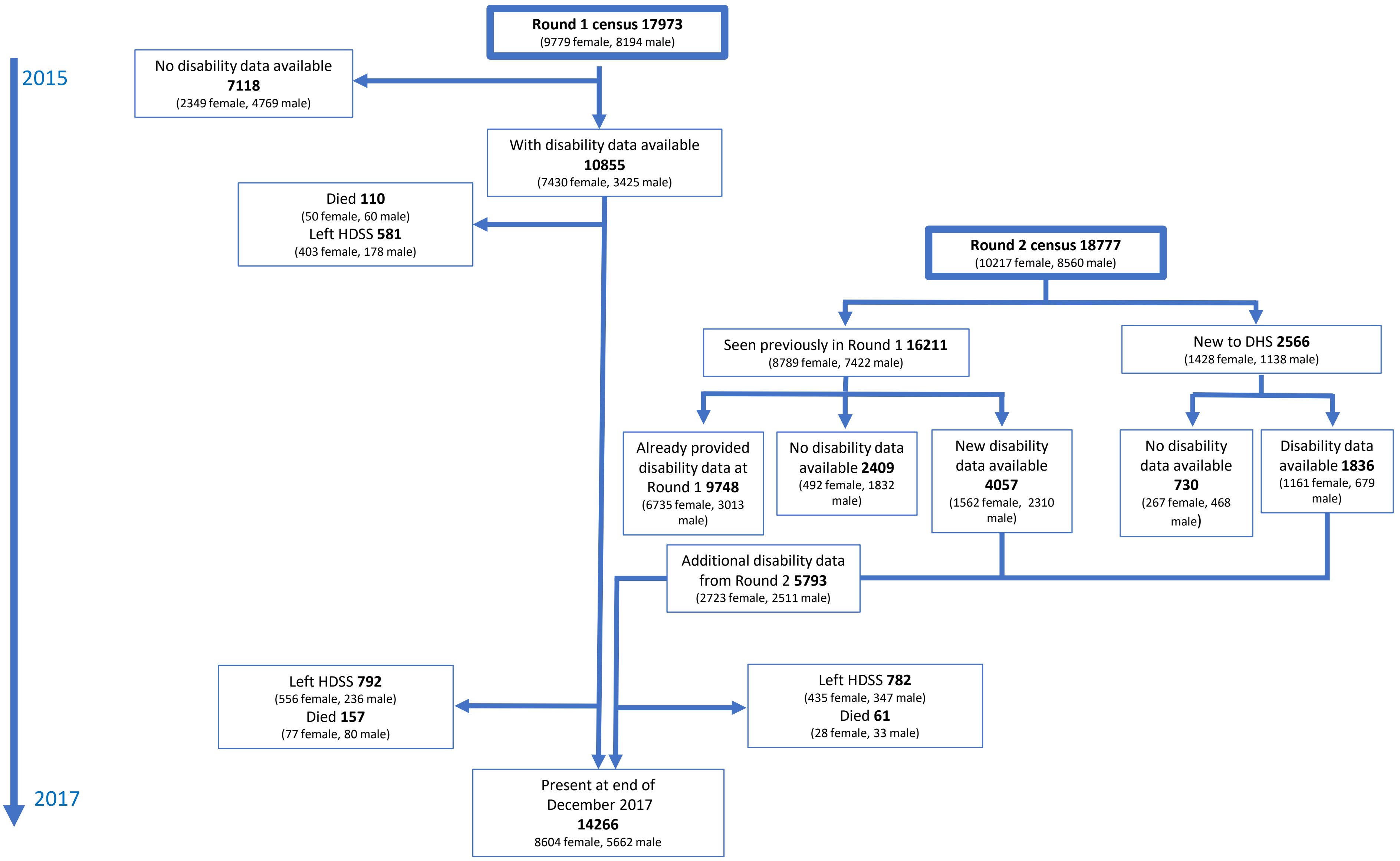
2015
2017
Round 1 census 17973
(9779 female, 8194 male)
No disability data available
7118
(2349 female, 4769 male)
With disability data available
10855
(7430 female, 3425 male)
Died 110
(50 female, 60 male)
Left HDSS 581
(403 female, 178 male)
Round 2 census 18777
(10217 female, 8560 male)
Seen previously in Round 1 16211
(8789 female, 7422 male)
New to DHS 2566
(1428 female, 1138 male)
Already provided disability data at Round 1 9748
(6735 female, 3013 male)
No disability data available 2409
(492 female, 1832 male)
New disability data available 4057
(1562 female, 2310 male)
No disability data available 730
(267 female, 468 male)
Disability data available 1836
(1161 female, 679 male)
Additional disability data from Round 2 5793
(2723 female, 2511 male)
Left HDSS 792
(556 female, 236 male)
Died 157
(77 female, 80 male)
Left HDSS 782
(435 female, 347 male)
Died 61
(28 female, 33 male)
Present at end of December 2017
14266
8604 female, 5662 male

Table S1. Baseline characteristics of census participants and study participants at Round 1

	Census			Study[1]		
	Women	Men	Total	Women	Men	Total
	n=9779	n=8194	n=17973	n=7430	n=3425	n=10855
**Age group**						
**18-34**	5142 (52.6)	4472 (54.6)	9614 (53.5)	3813 (51.3)	1544 (45.1)	5357 (49.4)
**35-44**	1874 (19.2)	1598 (19.5)	3472 (19.3)	1476 (19.9)	711 (20.8)	2187 (20.1)
**45-54**	1105 (11.3)	934 (11.4)	2039 (11.3)	814 (11.0)	443 (12.9)	1257 (11.6)
**55-64**	781 (8.0)	553 (6.7)	1334 (7.4)	606 (8.2)	306 (8.9)	912 (8.4)
**65-69**	280 (2.9)	192 (2.3)	472 (2.6)	213 (2.9)	105 (3.1)	318 (2.9)
**70-74**	197 (2.0)	142 (1.7)	339 (1.9)	173 (2.3)	101 (2.9)	274 (2.5)
**75-79**	200 (2.0)	154 (1.9)	354 (2.0)	164 (2.2)	96 (2.8)	260 (2.4)
**80+**	200 (2.0)	149 (1.8)	349 (1.9)	171 (2.3)	119 (3.5)	290 (2.7)
**Missing**	0	0	0	0	0	0
**Education level**						
**None**	379 (3.9)	104 (1.3)	483 (2.7)	296 (4.0)	69 (2.0)	365 (3.4)
**Some primary/completed primary**	6323 (65.1)	3961 (48.6)	10284 (57.6)	4975 (67.4)	1834 (53.7)	6809 (63.1)
**Some secondary/completed secondary**	2529 (26.1)	3442 (42.2)	5971 (33.4)	1757 (23.8)	1264 (37.0)	3021 (28.0)
**Tertiary**	477 (4.9)	649 (8.0)	1126 (6.3)	351 (4.8)	247 (7.2)	598 (5.5)
**Missing**	71	38	109	51	11	62
**Occupation**						
**not working**	1156 (12)	1497 (18.3)	2653 (14.9)	621 (8.4)	392 (11.5)	1013 (9.4)
**manual**	130 (1.3)	1086 (13.3)	1216 (6.8)	89 (1.2)	386 (11.3)	475 (4.4)
**farmer/fisherman**	7190 (74.3)	4639 (56.8)	11829 (66.3)	5716 (77.7)	2326 (68.1)	8042 (74.7)

**non-manual/ business/ professional**	1196 (12.4)	939 (11.5)	2135 (12)	928 (12.6)	310 (9.1)	1238 (11.5)
**Missing**	107	33	140	76	11	87
**Union status**						
**Not in a union**	3380 (34.6)	2635 (32.2)	6015 (33.5)	2339 (31.5)	869 (25.4)	3208 (29.6)
**In a union**	6391 (65.4)	5547 (67.8)	11938 (66.5)	5087 (68.5)	2553 (74.6)	7640 (70.4)
**Missing**	8	12	20	4	3	7
**BMI**						
**<18.5**	624 (7.2)	647 (9.6)	1271 (8.2)	482 (7.1)	298 (10.0)	780 (8.0)
**18.5-24.9**	5667 (65.0)	5434 (80.9)	11101 (71.9)	4344 (64.4)	2373 (79.8)	6717 (69.1)
**25-29.9**	1774 (20.3)	554 (8.2)	2328 (15.1)	1401 (20.8)	269 (9.0)	1670 (17.2)
**30+**	660 (7.6)	83 (1.2)	743 (4.8)	520 (7.7)	35 (1.2)	555 (5.7)
**Missing**	1054	1476	2530	683	450	1133
**Hypertension**						
**No hypertension**	6286 (85.8)	4639 (86.3)	10925 (86.0)	4871 (85.3)	2026 (82.4)	6897 (84.4)
**Hypertension**	1041 (14.2)	737 (13.7)	1778 (14.0)	837 (14.7)	434 (17.6)	1271 (15.6)
**Missing**	2452	2818	5270	1722	965	2687
**Diabetes**						
**No diabetes**	6400 (98.2)	4534 (98.2)	10934 (98.2)	4988 (98.3)	2057 (97.5)	7045 (98.1)
**Diabetes**	117 (1.8)	81 (1.8)	198 (1.8)	87 (1.7)	53 (2.5)	140 (1.9)
**Missing**	3262	3579	6841	2355	1315	3670
**HIV status**						
**Negative**	6804 (88.7)	5066 (91.0)	11870 (89.7)	5233 (88.4)	2206 (89.6)	7439 (88.7)
**Positive**	864 (11.3)	504 (9.0)	1368 (10.3)	686 (11.6)	257 (10.4)	943 (11.3)

For peer review only

**Missing**		2111	2624	4735	1511	962	2473
**Number of chronic diseases**							
	**0**	4689 (77.4)	3219 (78.6)	7908 (77.9)	3633 (77)	1393 (73.5)	5026 (76.0)
	**1**	1254 (20.7)	793 (19.4)	2047 (20.2)	1001 (21.2)	455 (24.0)	1456 (22.0)
	**2**	107 (1.8)	84 (2.0)	191 (1.9)	82 (1.7)	45 (2.4)	127 (1.9)
	**3**	5 (0.1)	2 (0.0)	7 (0.1)	4 (0.1)	2 (0.1)	6 (0.1)
**Missing**		3724	4096	7820	2710	1530	4240

1. Study participants defined as those with data on disability status

Table S2. Age-specific all-cause mortality rates of men with and without disability data[1]

	With disability data			Without disability data		
**Age group**	**Number of deaths**	**Person-years**	**Rate per 1000 PY**	**Number of deaths**	**Person-years**	**Rate per 1000 PY**
**18-49**	56	10198	5.49 (4.23-7.14)	29	5502	5.27 (3.66-7.86)
**50-69**	56	2207	25.37 (19.52-32.97)	18	3623	36.23 (22.82-57.50)
**70+**	61	972	62.74 (48.81-80.63)	12	65	184.23 (104.63-324.40)

1. p value for heterogeneity between men with and without disability data = 0.94

Table S3. Poisson regression analysis of effect of graded levels of self-reported disability on all-cause mortality

	Number of deaths	Person-years	Model 1: adjusted for age only[1]	Model 2: adjusted for age, sex, & occupation[2]	Model 3: adjusted for age, sex, occupation, BMI, hypertension, diabetes, & HIV status[3,4]

For peer review only

			RR (95% CI)	p- value[5]	RR (95% CI)	p- value[5]	RR (95% CI)	p- value[5]
**Any disability**								
**No difficulty**	48	13248	1		1		1	
**Some difficulty**	82	7537	1.31 (0.87-1.99)	0.12	1.51 (0.99-2.29)	0.10	1.50 (0.99-2.28)	0.09
**A lot of difficulty/ can't do at all**	50	1937	1.71 (1.02-2.86)		1.67 (0.98-2.85)		1.71 (1.01-2.90)	

1. Adjusted for age (continuous variable); excluding participants who were missing data on occupation, BMI, hypertension, diabetes, or HIV to allow comparison with Models 2 and 3
2. Adjusted for age (continuous variable), sex (male/female), and occupation (not working, manual work, farming/fishing, non-manual work); excluding participants who were missing data on BMI, hypertension, diabetes, or HIV to allow comparison with Models 1 and 3
3. Adjusted for age (continuous variable), sex (male/female), occupation (not working, manual work, farming/fishing, non-manual work), BMI (<18.5kg/m^2, 18.5-24.9kg/m^2, 25-29.9kg/m^2, 30+kg/m^2), hypertension (defined as a measured BP >140/90 or on antihypertensive medication), diabetes (defined as fasting blood sugar >7.0 or previous diagnosis of diabetes), and HIV status (defined as a positive HIV test or diagnosis ever)
4. Likelihood ratio test for heterogeneity in the association of disability and all-cause mortality by these factors: BMI p=0.08; hypertension p=0.67; diabetes p=0.41; HIV p=0.88
5. P value is a likelihood ratio test for difference

Table S4. Poisson regression analysis of effect of graded levels of self-reported disability on all-cause mortality by hypertension status (including unknown)[1,2]

	**No hypertension**			**Hypertension**			**Unknown hypertension status**		
	Deaths	Person-years	RR (95% CI)	Deaths	Person-years	RR (95% CI)	Deaths	Person-years	RR (95% CI)
**No disability**	48	14009	1	9	1286	1	27	7099	1
**Some difficulty**	59	6719	1.48 (0.99-2.20)	34	1790	1.72 (0.82-3.61)	30	2217	1.98 (1.14-3.41)
**A lot of difficulty / can't do at all**	30	1461	1.93 (1.16-3.20)	40	844	2.27 (1.07-4.81)	51	588	5.10 (2.94-8.83)

1. All models are controlled for age (continuous variable), sex, occupation (not working, manual work, farming/fishing, non-manual work)
2. p-value for heterogeneity = 0.03

For peer review only

STROBE Statement—checklist of items that should be included in reports of observational studies

	Item No	Recommendation	
**Title and abstract**	1	(*a*) Indicate the study's design with a commonly used term in the title or the abstract	Page 1
		(*b*) Provide in the abstract an informative and balanced summary of what was done and what was found	Page 2
**Introduction**			
Background/ rationale	2	Explain the scientific background and rationale for the investigation being reported	Page 4
Objectives	3	State specific objectives, including any prespecified hypotheses	Page 5
**Methods**			
Study design	4	Present key elements of study design early in the paper	Pages 5-6
Setting	5	Describe the setting, locations, and relevant dates, including periods of recruitment, exposure, follow-up, and data collection	Pages 5-6
Participants	6	(*a*) *Cohort study*—Give the eligibility criteria, and the sources and methods of selection of participants. Describe methods of follow-up<br>*Case-control study*—Give the eligibility criteria, and the sources and methods of case ascertainment and control selection. Give the rationale for the choice of cases and controls<br>*Cross-sectional study*—Give the eligibility criteria, and the sources and methods of selection of participants	Page 6
		(*b*) *Cohort study*—For matched studies, give matching criteria and number of exposed and unexposed<br>*Case-control study*—For matched studies, give matching criteria and the number of controls per case	
Variables	7	Clearly define all outcomes, exposures, predictors, potential confounders, and effect modifiers. Give diagnostic criteria, if applicable	Pages 5-6
Data sources/ measurement	8*	For each variable of interest, give sources of data and details of methods of assessment (measurement). Describe comparability of assessment methods if there is more than one group	Pages 5-6
Bias	9	Describe any efforts to address potential sources of bias	Pages 5-6
Study size	10	Explain how the study size was arrived at	n/a
Quantitative variables	11	Explain how quantitative variables were handled in the analyses. If applicable, describe which groupings were chosen and why	Page 6
Statistical methods	12	(*a*) Describe all statistical methods, including those used to control for confounding	Page 6
		(*b*) Describe any methods used to examine subgroups and interactions	Page 6
		(*c*) Explain how missing data were addressed	Page 6
		(*d*) *Cohort study*—If applicable, explain how loss to follow-up was addressed<br>*Case-control study*—If applicable, explain how matching of cases and controls was addressed<br>*Cross-sectional study*—If applicable, describe analytical methods taking account of sampling strategy	Page 6
		(*e*) Describe any sensitivity analyses	Page 6

For peer review only

Continued on next page

For peer review only

**Results**			
Participants	13*	(a) Report numbers of individuals at each stage of study—eg numbers potentially eligible, examined for eligibility, confirmed eligible, included in the study, completing follow-up, and analysed	Figure 1
		(b) Give reasons for non-participation at each stage	Figure 1
		(c) Consider use of a flow diagram	Figure 1
Descriptive data	14*	(a) Give characteristics of study participants (eg demographic, clinical, social) and information on exposures and potential confounders	Table 1
		(b) Indicate number of participants with missing data for each variable of interest	Table 1
		(c) *Cohort study*—Summarise follow-up time (eg, average and total amount)	Page 8
Outcome data	15*	*Cohort study*—Report numbers of outcome events or summary measures over time	Page 8
		Case-control study—Report numbers in each exposure category, or summary measures of exposure	
		Cross-sectional study—Report numbers of outcome events or summary measures	
Main results	16	(*a*) Give unadjusted estimates and, if applicable, confounder-adjusted estimates and their precision (eg, 95% confidence interval). Make clear which confounders were adjusted for and why they were included	Table 2
		(*b*) Report category boundaries when continuous variables were categorized	
		(*c*) If relevant, consider translating estimates of relative risk into absolute risk for a meaningful time period	
Other analyses	17	Report other analyses done—eg analyses of subgroups and interactions, and sensitivity analyses	Supplementary tables
**Discussion**			
Key results	18	Summarise key results with reference to study objectives	Page 10
Limitations	19	Discuss limitations of the study, taking into account sources of potential bias or imprecision. Discuss both direction and magnitude of any potential bias	Page 11-12
Interpretation	20	Give a cautious overall interpretation of results considering objectives, limitations, multiplicity of analyses, results from similar studies, and other relevant evidence	Page 12
Generalisability	21	Discuss the generalisability (external validity) of the study results	Page 12
**Other information**			
Funding	22	Give the source of funding and the role of the funders for the present study and, if applicable, for the original study on which the present article is based	Page 14

*Give information separately for cases and controls in case-control studies and, if applicable, for exposed and unexposed groups in cohort and cross-sectional studies.

Note: An Explanation and Elaboration article discusses each checklist item and gives methodological background and published examples of transparent reporting. The STROBE checklist is best used in conjunction with this article (freely available on the Web sites of PLoS Medicine at http://www.plosmedicine.org/, Annals of Internal Medicine at http://www.annals.org/, and Epidemiology at http://www.epidem.com/). Information on the STROBE Initiative is available at www.strobe-statement.org.

For peer review only

For peer review only - http://bmjopen.bmj.com/site/about/guidelines.xhtml
